# Maternal flexibility in Galápagos sea lions mitigates climate impacts but not population decline

**DOI:** 10.1093/beheco/arag107

**Published:** 2026-09-01

**Authors:** Alexandra K Childs, Claire Stainfield, Svenja Stoehr, Sean D Twiss, Oliver Krüger

**Affiliations:** Department of Animal Behaviour, Faculty of Biology, Bielefeld University, Konsequenz 45, Bielefeld 33615, Germany; School of Natural and Social Sciences, Scotland’s Rural College (SRUC) Aberdeen Campus, Craibstone Estate Craibstone Drive, Aberdeen AB21 9TR, United Kingdom; Department of Animal Behaviour, Faculty of Biology, Bielefeld University, Konsequenz 45, Bielefeld 33615, Germany; Department of Biosciences, Durham University, Stockton Road, Durham, DH1 3LE, United Kingdom; Department of Animal Behaviour, Faculty of Biology, Bielefeld University, Konsequenz 45, Bielefeld 33615, Germany; Joint Institute for Individualisation in a Changing Environment (JICE), Bielefeld University and University of Münster, Konsequenz 45, Bielefeld 33615, Germany

**Keywords:** maternal care, behavioral plasticity, offspring survival, reproductive behavior, Pinnipedia

## Abstract

The Galápagos sea lion is an endangered marine apex predator that has experienced a 50% to 75% population decline over the last 50 yr. Using 21 yr of data, we present the first long-term analysis of pupping trends and pup survival in this species, revealing how extreme climate oscillations may shape population dynamics and future trends. We found that the Caamaño colony has been in decline since monitoring began in 2003, with the rate accelerating significantly after the 2010 reproductive period. This pattern cannot be explained by climate effects or reduced female fecundity but is more likely the result of decreasing recruitment due to lowered juvenile survival. Although there has been no overall change in median pupping point date, there has been a significant shortening of the duration of the pupping period since the acceleration of the population decline. We found that maternal foraging strategy has a significant effect on pupping timing, and its interaction with resource availability causes further interannual variation in timing. Male pups are born significantly earlier suggesting sex differences in gestational length or implantation. Although climate does affect pup survival to yearling age, there has been no temporal change in survival over the study period. Body condition at birth and juvenile presence within the colony have a positive impact on pup survival. Our results reveal that Galápagos sea lions exhibit resilience to the extreme climate variability of their environment through reproductive plasticity around parturition and that their continued decline is likely driven by reduced recruitment and increased juvenile mortality.

## Teaser

This first long-term (21 year) analysis of pupping trends in Galápagos sea lions reveals that mothers with specific foraging strategies exhibit resilience to extreme climate variability through reproductive plasticity around parturition. Nevertheless, the study colony has been declining since monitoring began in 2003 (-3% annually), a decline which accelerated after 2010 (-8.4% annually). Thus, female adaptability does not fully protect the population, with continued decline likely driven by reduced recruitment and increased juvenile mortality.

## Introduction

Mammals exhibit a diverse range of reproductive behaviors to maximize offspring fitness and survival (Clutton-Brock 1991; Kölliker et al. 2012; Heldstab et al. 2021) as well as mitigating the metabolic overhead to the primary caregiver (Trillmich 2010). The timing of parturition may be influenced by climate-driven variation in resource availably (Bronson 2009; Heldstab et al. 2018; Marley et al. 2022; Brogi et al. 2022), maternal age (Hastings and Jemison 2016; Bull et al. 2021), predation pressure (O’Donoghue and Boutin 1995; Michel et al. 2020), social structure (Sapolsky 2005; Silk et al. 2009; Clutton-Brock 2021), intraspecific competition (Hodge et al. 2011), and population density (Stewart et al. 2005; Richard et al. 2014; Williams et al. 2014), all of which may also impact offspring survival (Tecot 2010; da Silva et al. 2023). Early maternal investment is a key determinant of dependent offspring growth and survival. Reduced investment, often associated with poor maternal nutritional condition, has been linked to lower survival rates (Skibiel and Hood 2015; Avery and Zinn 2023; Lamb et al. 2023). Maternal age has been shown to positively influence offspring survival, with older, more experienced females generally producing more viable offspring and achieving higher reproductive success (LaSharr et al. 2025; Ravindran et al. 2025). These maternal effects interact with climate and resource availability, further shaping reproductive outcomes (English et al. 2014; Caton and Hess 2019; Costa and Valenzuela-Toro 2021).

Marine mammals are dependent on annual cycles of ocean productivity driven by weather patterns and upwellings, making them important indicators of ocean health and ecosystem change (Moore 2008; Plön et al. 2024). Some species may travel thousands of kilometers on an annual basis to take advantage of seasonal resource abundance (Staniland et al. 2018; Condit et al. 2022). Among marine mammals, pinnipeds face the challenge of breeding on land (or ice) while being dependent on ocean productivity (Pomeroy 2011; Avery and Zinn 2023). As a result, the timing of breeding cycles has evolved so mothers can more easily support dependent offspring, who are usually highly energetically demanding (Forcada and Staniland 2009; Henderson et al. 2014; Geeson et al. 2022).

Several pinniped species exhibit interannual variation in pupping timing that has been linked to the impact of climatic variability on resource availability (Boyd 1996; Forcada et al. 2005; Soto et al. 2006; Geeson et al. 2022). Overall shifts in peak pupping point have been identified in South American sea lions (*Otaria flavescens*, Soto et al. 2004), harbor seals (*Phoca vitulina*, Jemison and Kelly 2001; Osinga et al. 2012; Cordes and Thompson 2013), and gray seals (*Halichoerus grypus*, Bull et al. 2021), which have been attributed to climate factors (Pelayo-González et al. 2021). At the intraspecific level, variation in pupping phenology has been linked to maternal age (Jemison and Kelly 2001; Maniscalco et al. 2006; Hastings and Jemison 2016; Bull et al. 2021), maternal nutritional health and its association with the embryonic diapause duration (Gales et al. 1997; Bowen et al. 2003; Reijnders et al. 2010; Osinga et al. 2012), and local differences in resource availability (Pitcher et al. 2001; Maniscalco 2014), which may be further impacted by foraging strategy (Schwarz et al. 2022). In addition to environmental variability, intrinsic population factors can also shape reproductive outcomes. Population density can influence survival in various ways; higher densities have been associated with benefits, such as enhanced defense against predation on offspring (Lydersen and Kovacs 1999; Laroche et al. 2008; Nagel et al. 2021, Berthelsen et al. 2026) but also with costs, including increased resource competition (Breed et al. 2013).

Within pinnipeds 2 breeding strategies have evolved. The majority of *phocids* (true seals) are capital breeders, where mothers remain with their neonate offspring for a short but intense period of dependency, relying on stored energy reserves until weaning (Costa and Maresh 2022). The timing of pupping in *phocids* therefore often coincides with the availability of suitable breeding habitats, and weaning coincides with an annual local increase in productivity, improving the survival probability of weaners during a sensitive skill development stage (Jemison and Kelly 2001; Stephens et al. 2014; Womble et al. 2021; Robinson and Pomeroy 2022). In contrast, *otariids* (eared seals), such as the Galápagos sea lion (GSL) (Z*alophus wollebaeki*), practice income breeding where mothers continue to forage throughout their offspring dependency allowing for a prolonged lactation period (Schulz and Bowen 2004; McHuron et al. 2023). As a result, lactating females are limited in foraging trip duration, and breeding colonies are usually found in areas that provide reliable access to prey for both nursing mother and newly weaned offspring (Burkanov et al. 2011; Champagne et al. 2013). Many offspring develop foraging skills while still nursing (Lowther and Goldsworthy 2012), leading to high intraspecific variation in weaning age (Gibbens and Arnould 2009; Hastings et al. 2021; Childs et al. 2026a). Consequently, parturition—rather than weaning—is timed to coincide with high oceanic productivity (Soto et al. 2006; Wall et al. 2023).

GSL are an endangered pinniped endemic to the Galápagos archipelago. As a Pacific equatorial species, they are subjected to the El Niño Southern Oscillation (ENSO) phenomenon which directly impacts prey abundance for these central-place foragers (Trillmich and Limberger 1985; Soto et al. 2004). They exhibit 3 distinct foraging strategies (benthic, pelagic, and night divers; Villegas-Amtmann et al. 2008; Schwarz et al. 2021), which have varying foraging efficiencies and distinct sensitivities and responses to environmental change (Blakeway et al. 2021; Schwarz et al. 2022). GSLs are currently the only pinniped known to hunt cooperatively, suggesting that social links extend beyond mother and dependent offspring pairings (Páez-Rosas et al. 2020; De Roy et al. 2021; Childs et al. 2026a). Maintaining a healthy population size may therefore be critical for colony social stability and survival success (Zenth et al. 2021; Nagel et al. 2022; Berthelsen et al. 2026). Consistent with these GSLs are highly gregarious and tactile, with strong site fidelity forming an important structural component in an individual’s social interaction space (Wolf et al. 2007). GSL females return to the same home ranges over multiple years, increasing the likelihood of repeat interactions with conspecifics over a lifetime (Wolf and Trillmich 2007).

GSL females show high reproductive asynchronicity (Villegas-Amtmann et al. 2009) within a protracted pupping period (September–January, Pörschmann et al. 2010). Females may influence birth interval through mate choice (Pörschmann et al. 2010) or delayed entry to estrus after parturition (Trillmich 1986). On average, GSL give birth to a single pup every 2 yr from ∼6 yr of age (Kalberer et al. 2018). After a 7-d perinatal period (Trillmich 1986), lactating mothers alternate between foraging trips (2.5 to 8 d) and providing maternal care (Villegas-Amtmann et al. 2008). Offspring are entirely dependent on maternal provisioning for the first year of life after which they begin to develop foraging skills (Jeglinski et al. 2012; Piedrahita et al. 2014), a skill development stage which may contribute to the high variability in weaning age (1.5 to 4.5 yr, Childs et al. 2026a). This flexibility in the duration of maternal care likely contributes to the maternal buffering previously identified in response to developmental and survival fluctuations associated with ENSO and therefore prey availability (Kraus et al. 2013).

GSLs have suffered a 50% population decline over the last 40 yr (Krüger et al. 2021; Riofrío-Lazo and Páez-Rosas 2023). Current threats contributing to the decline include climate fluctuations, predation, and human interactions—such as tourism and fisheries conflict—all of which can lead to behavioral changes, disease, injury, and death (Denkinger et al. 2015; Páez-Rosas et al. 2021). Tourism depends on the archipelago’s marine fauna, and as a highly charismatic species, GSLs play an important role in the industry. However, population trends across the archipelago vary; some colonies are growing, whereas others remain stable or are declining (Riofrío-Lazo et al. 2017). Such disparate trends could result in misconceptions of the overall population health. Effective conservation may therefore require colony-specific monitoring, particularly for colonies exposed to greater anthropogenic pressures (Riofrío-Lazo et al. 2017).

This study utilizes data from a long-term demographic study of the Isla Caamaño colony to examine trends in GSL pupping behavior. Caamaño is home to formally one of the largest breeding colonies in the Santa Cruz area and is located within 2 km of the archipelago’s capital, Puerto Ayora, and busiest harbor; it therefore provides a unique insight into the breeding resilience of this species over multiple decades. Specifically, we aim to (i) evaluate pupping trends over 21 yr to assess the future health of the study colony, (ii) characterize temporal variation in the median pupping point date (MPP) and reproductive timing both at a population and an individual level, and (iii) quantify yearling survival. For each of these aims, we investigate the influence of climatic variability, population density, maternal investment, and maternal characteristics on pupping behavior and yearling survival. Given the ongoing decline of the GSL population (Krüger and Trillmich 2026) and the increasing intensity and frequency of El Niño events under climate change (Cai et al. 2014), understanding their influence on pupping behavior, reproductive success, and yearling survival at a long-term scale is essential for informing successful conservation management to safeguard this endangered species. However, our study also highlights the importance of exploring colony- and life-stage-specific dynamics to inform more effective and tailored conservation strategies.

## Materials and methods

### Data collection

**Figure 1:**
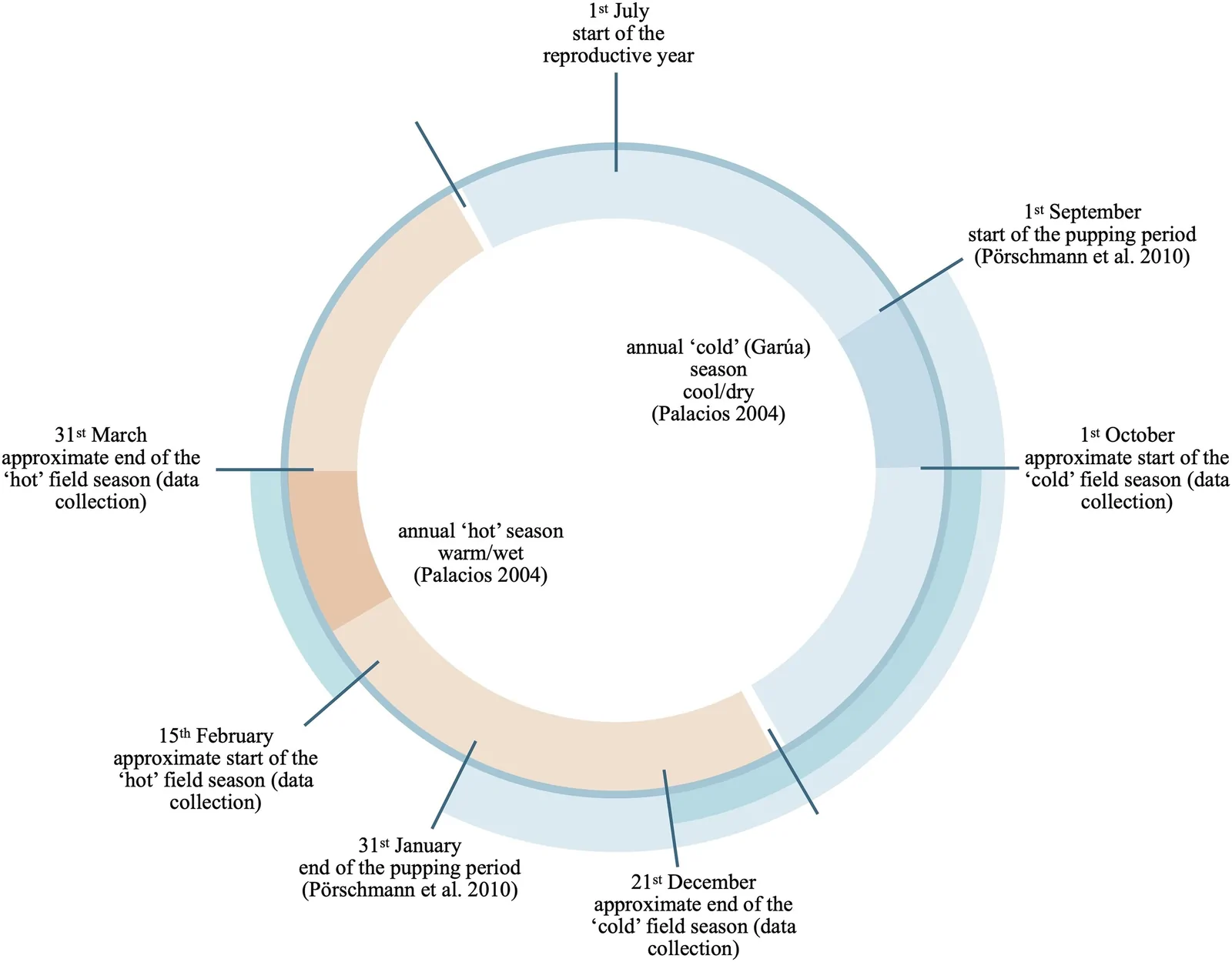
The GSL reproductive year as defined in this study. Shaded areas on the exterior of the circle represent the pupping period and annual field seasons during which data were collected. Shaded areas on the interior of the circle represent the annual cold and hot seasons of the Galápagos (Palacios 2004), which are independent of ENSO events, and the darker shaded areas of these seasons represent the months when temperatures reach their minimum and maximum.

Data were collected as part of a long-term demographic study between February 2003 and April 2023 from a closed colony of GSLs (Trillmich et al. 2016) located on Isla Caamaño (0°45′S, 90°16′W), off the southern coast of Santa Cruz, Galápagos. Annual field work was split over 2 periods that were timed to cover the bulk of the pupping period (“cold” field season, ∼8 to 10 wk in October–December, Pörschmann et al. 2010) and soon after pupping has ended (“hot” field season, ∼2 to 3 wk in February–April) (Fig. 1). “Cold” (locally known as Garúa) and “hot” seasons refer to the annual seasonal changes in temperature within the archipelago, which may be exacerbated by the ENSO (Fig. 1) (Palacios 2004). Data collection during the hot season was only implemented after 2005. Due to coronavirus disease 2019 (COVID-19) in 2020, no cold season took place, and data collection was restricted to an extremely short, late hot season. Data were grouped by reproductive year analogously to Childs et al. (2026b). However, due to the presence of 4 extremely early estimated birth dates (see Supplementary material 2.1) recorded in late July, the start and end dates were shifted by 1 mo to July–June, so that all offspring were assigned to the correct cohort for annual pupping success and survival analysis (Fig. 1). Reproductive years are referred to forthwith by the calendar year (Gregorian) in which they began. All individuals that are born on and use the islet regularly were uniquely marked allowing for the collection of detailed life-history data (Wolf and Trillmich 2007; Meise et al. 2014). All marked individuals were assigned a date of birth (DoB), which was categorized according to their accuracy (Supplementary material 2.1). Pups were handled for the first time when their mother left for her first postpartum foraging trip to avoid disrupting the mother–pup bond. Length (tip of the nose to tip of the tail [cm]) and mass (kg) measurements, used to calculate body condition, were taken during handling using a standardized ruler placed alongside the pup and a digital scale (see Mueller et al. 2011).

**Figure 2:**
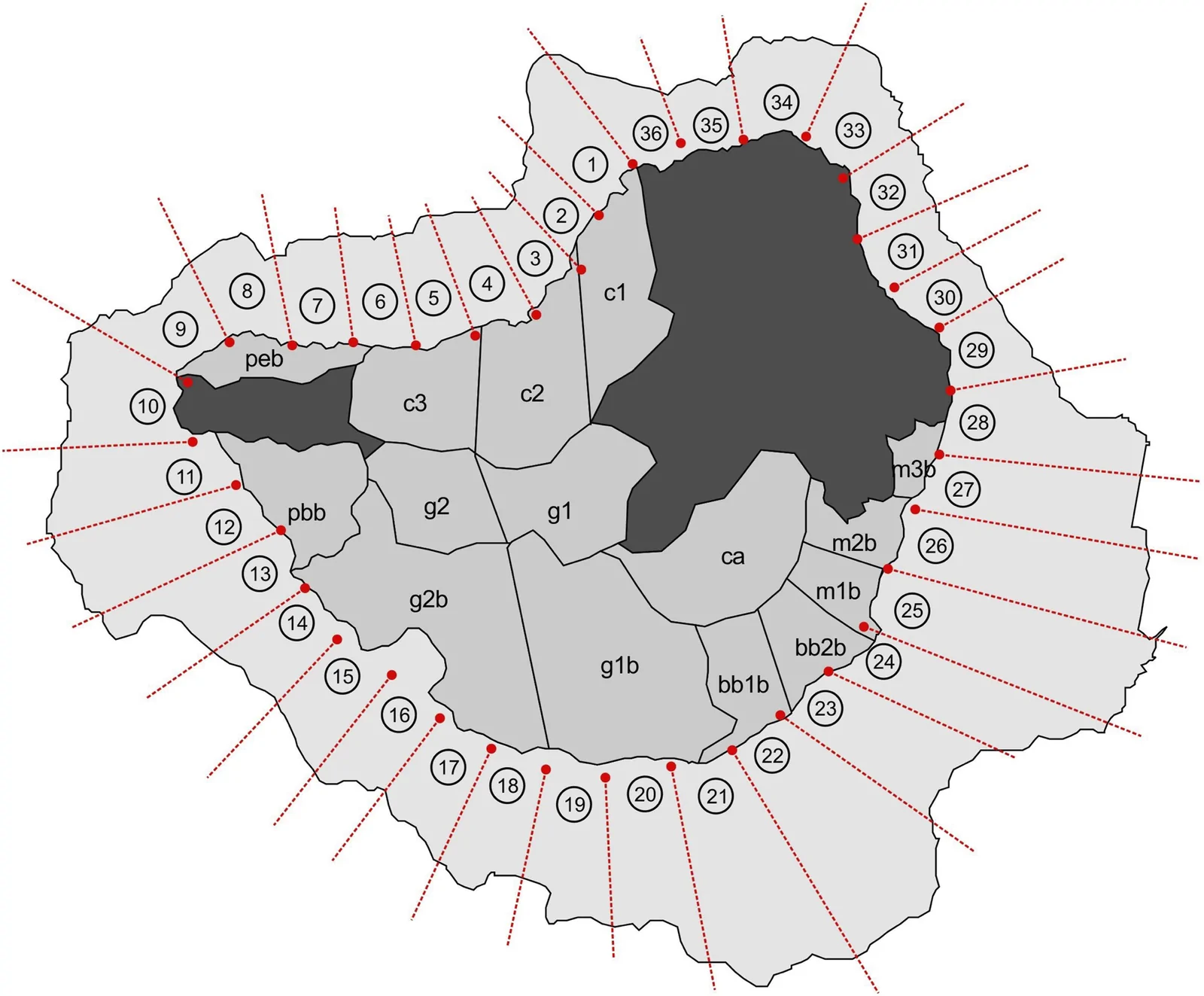
Artificial division of the islet in use since 2007 (Zenth et al. 2021). Light gray areas are coastal sites with direct access to the sea; medium gray areas are interior sites that are either covered in vegetation or are bare, dusty ground. Dark gray zones are characterized by extremely uneven ground and are made up of sharp rocks and/or covered in very thick vegetation that make them inaccessible to both sea lions and researchers.

The islet is divided into 51 sites (Fig. 2); the current map layout has been in use since 2007. Costal sites are ∼10 m wide at the point furthest from the ocean and vary in length depending on the tidal state; interior sites are of varying fixed sizes and are separated by natural features such as vegetation type. Extremely popular interior sites are subdivided for assessing finer-scale site use. Identification rounds covering the entire islet were conducted 2 to 5 times a day during field seasons, for 40 to 149 observation days per year (effort varied according to concurrent projects and the number of field seasons). During identification rounds, the ID and location of every individual on the islet who could be identified were recorded. Census rounds were conducted once every 2.5 d where the ID and location of all individuals, including those who could not be identified, were recorded (see Trillmich et al. 2016).

### Data treatment

For each reproductive year, the peak pupping point was calculated as the median pupping point (MPP), when 50% of the total births had occurred (Soto et al. 2004; Geeson et al. 2022). Duration of the pupping period was calculated as the number of days between the birth of the year’s first and last pup. Dates of birth (DoBs) with an uncertainty of >2 wk were excluded from the calculation of both the median and pupping duration. DoBs with a certainty of ±2 wk are collectively referred to as “known” births (Supplementary material 2.1). When assessing temporal trends in annual births and yearling survival, the data set was expanded to include births with a greater level of uncertainty (6 mo, Supplementary material 2.1) but could still be correctly assigned to their cohort of birth. To understand inter and intra-annual variation in the timing of births, all MPPs and individuals were assigned a Julian day of birth, adjusted to follow the reproductive year. We calculated effort as the total number of observation days and the presence or absence of a second field season (Supplementary material 2.2). To understand the impact density may have on pupping success, behavior, and yearling survival, the mean annual number of adult males, adult females, and adults of an unknown sex (≥5 yr of age and considered sexually mature), as well as juveniles (1 to 5 yr of age, who may or may not have weaned) were calculated using census counts. The annual maximum number of females observed on the islet, and the total number of recorded births was also taken. Due to offspring’s complete dependency on maternal milk during their first year, slow physiological development (Jeglinski et al. 2012; Piedrahita 2015), and high site fidelity (Wolf and Trillmich 2007), pups were assumed to have survived to yearling age if they were observed on the islet the following reproductive year.

To understand the role of early maternal investment in yearling survival, in the context of climate, pup body condition at birth (≤10 d) was calculated using scaled mass index (SMI) (Peig and Green 2009). Where multiple measurements were available, those taken at the youngest age were used. As climate conditions are known to more acutely impact pelagic foragers (Schwarz et al. 2022), where it was known, we included maternal foraging strategy as a potential driver of differences in maternal investment. Benthic and pelagic strategies were classified according to dive data obtained using time-depth recorders (Schwarz et al. 2021), and vibrissae length measured from photographs (Stoehr et al. 2026). Because these strategies remain stable over time and climate conditions (Stoehr et al. 2026), a female’s foraging classification was associated with all births registered to her within the dataset (*n* = 79). Maternal age in years was calculated for mothers with a known and estimated DoB (±6 mo). As GSL females are philopatric and frequently give birth at the same site for successive offspring, birth site was used to assess if parturition location may be a strategy to improve offspring survival (van Noordwijk et al. 2012; Supplementary material 2.2.). Similarly, we used birth site conspecific density to understand any social benefits in parturition site (Zenth et al. 2021; Supplementary material 2.2). Due to the current division of the islet (Fig. 2) only being implemented from 2007, to ensure that data were comparable these latter 2 metrics were only calculated for offspring born from 2007 onward.

Monthly temperature anomaly data were obtained from the US National Oceanic and Atmospheric Administration (NOAA 2025) to assess the impact of ENSO on the pupping period and yearling survival. In order to disentangle the possible effects of resource availability and annual seasonal temperature variations on parturition, prepartum, and postpartum maternal investment, for each year during the study we calculated separately the mean anomaly for the partum year (July–June, the year of birth), the first 6 mo of the partum year (July–December), the prepartum year (July–June, prior to the partum year), and last 6 mo of the prepartum year (January–June, prior to the partum year), resulting in 4 values per year (Fig. S1). Our biological reasoning behind each of these values can be found in Supplementary material 2.2 (Fig. S1). Oceanic Niño Index (ONI) categories (“El Niño,” “Intermediate,” and “La Niña”) as defined by NOAA (2025) were assigned according to the mean anomaly to provide a broad overview of resource availability (Supplementary material 2.2). In all statistical testing, the reference category for ONI was set to “Intermediate.” Local sea surface temperature (SST) is known to directly impact the success of certain foraging strategies (Schwarz et al. 2021). We therefore obtained daily SST measurements from the nearest meteorological center, located in Puerto Ayora, Santa Cruz (Charles Darwin Foundation 2025), and calculated the mean SST for the 6-mo prior and 6-mo post the start of each reproductive year.

### Statistical analysis

#### Change in pupping numbers

A generalized additive model (GAM) was used to estimate the onset of decline, hereafter referred to as the “breakpoint,” in pupping activity. To understand if the decline was associated with the number of females in the colony or indicative of a change in fecundity, we plotted the slopes of the number of births and maximum count of females per year. We then tested for slope differences using a linear model (LM) with year as an interaction to account for any hidden cohort effects. Due to high data dispersion, we used a negative binomial (NB) generalized LM (GLM) (Lindén and Mäntyniemi 2011) to calculate the rate of decline in births while adjusting for effort (*n* observation days, see Supplementary material 2.2); and a second for the rate of births per female adjusting for the number of females (Supplementary material 2.3) over the study period. To allow for any changes in slope before and after the onset of decline, the breakpoint was included as a hinge term. We extrapolated the trend of the birth rate from after the breakpoint until 2030. Year was added as a fixed effect to control for any hidden cohort effects. Because the median year corresponded to the breakpoint, year was mean-centered on the first year of decline (*t*_0_).

To understand temporal trends in annual sex ratios, we tested for significant differences between the proportion of males and females born each season using a χ^2^ test. Trends in the ratios were tested for using a binomial GLM with year included as a continuous predictor (Table S2). To ascertain the impact of the climate and colony density on the number of births recorded annually within the colony, we used NB GLMs (Table S2). For density, we tested the mean counts across sex and age categories for each reproductive year (Table S1). Given the high variation in births “pre” and strong decline “post” the breakpoint, when testing climate and density covariates, each value was tested individually as fixed effects in 3 variations of the base NB GLM: (i) covariate, (ii) covariate + breakpoint, and (iii) covariate × breakpoint. Breakpoint was also tested independently as a categorical variable to better understand effect changes before and after the onset of the decline. Anomaly and density counts were centered for ease of intercept interpretation. Covariate selection and model formulation for the final NB GLM were guided by significance and corrected Akaike information criterion (AICc) due to the small sample size and number of parameters (Symonds and Moussalli 2011).

#### Pupping period

We calculated the overall MPP using all known births recorded within the Caamaño colony during the data collection period (*n* = 1,998). To better understand the temporal distribution of births, we plotted the pupping trend across a year with a 95% confidence interval (CI) and calculated the windows of when 50% and 95% of all pups were born across the study period. The effect of effort (*n* field seasons), the number of births, duration of the pupping period, climate (Table S1), colony density (Table S1), and the annual average maternal age at parturition on MPP was tested in a step-by-step process using LMs (Table S3). The reproductive year 2020 was removed from all analyses due to the extremely short and late field season resulting from COVID-19 restrictions. To aid in the interpretation of any maternal age effects caused by missing data, we calculated the proportion of mothers of an unknown age for each year. All climate and density covariates were tested individually as fixed effects in 3 variations of the LM being built upon: (i) covariate, (ii) covariate + breakpoint, and (iii) covariate × breakpoint. The inclusion of covariates in the final LM was guided by significance and AICc (Symonds and Moussalli 2011).

To investigate temporal trends and changes in the duration of the pupping period, we used LMs and, in a step-by-step process, tested the effect of effort (*n* field seasons), the number of births, MPP, climate (Table S1), colony density (Table S1), and the annual average maternal age at parturition (Table S4). Prior to this, preliminary analysis was undertaken to assess the reliability of effort in its selected form (Supplementary material 2.3). The reproductive year 2020 was removed from all analysis due to the extremely short and late field season resulting from COVID-19 restrictions. The inclusion of covariates in the final LM was guided by significance and AICc. Due to GSL having such a long pupping period, better understand variation in the timing of individual births, we fitted linear mixed-effects models predicting the Julian day of birth (known DoB) as a function of scaled effort, breakpoint, sex, climate, birth SMI, maternal age at parturition and maternal foraging strategy, with year as a random effect (Table S5). As not all data were available for all individuals, data were subset and variable informativeness assessed by model comparison for each relevant subset (Burnham and Anderson 2002). Given that resource availability is impacted by SST, which in turn impacts foraging strategies differently (Schwarz et al. 2022), we investigated if this niche choice (Trappes et al. 2022) influences the timing of offspring births. SST was included in the model as an interaction effect as this has been shown to be the most parsimonious approach for modeling climate effects (Schwarz et al. 2022). We tested separately the mean of the 6 mo prior and post the start of the reproductive year as a proxy for late gestational investment and early milk provisioning, respectively. Inclusion of covariates in the final model was assessed in a step-by-step process and guided by significance and Bayesian information criterion (BIC) (Schwarz 1978; Vrieze 2012). In cases where BIC and statistical significance did not agree, we favored the simpler version of the model (Schwarz 1978; Chowdhury and Turin 2020).

#### Yearling survival

We examined yearling survival of pups born between 2003 and 2022. To assess overall survival, we calculated the proportion of yearling survivors for the data collection period and each year. We used logistic regression models (LRMs) to assess effects on yearling survival probability, including breakpoint, climate, sex, effort (observation days), colony density, MPP, Julian day of birth, birth site (as a factor), conspecific density at birth site, birth SMI, and maternal age (Table S5). When testing the effect of effort, we used the value associated with the individual’s yearling year, ie when they were identified as having survived. When testing climate and density variables, we used the annual mean values of the individual’s birth year (Table S1). For birth site, the site (Fig. 2) with the proportion of yearling survivors closest to the overall survival proportion was set as the reference. As not all information was available for all individuals, variables were tested in a step-by-step manner where at each stage we compared model fit with and without that variable based on the same sample. The effect of a variable was guided by BIC, which also informed model selection and parameter inclusion in all LRMs. Where there was contradiction between significance results and BIC, the simpler version of the model was favored (Schwarz 1978; Chowdhury and Turin 2020).

To further assess the impact of effort on the accuracy of our results, all statistical analyses were duplicated and run using only DoB that were directly observed (Supplementary material 2.1). These results are reported in full in the Supplementary Material Results. All data manipulation and analysis were carried out in RStudio 2024.9.0.375 (Posit team 2024) using R (R Core Team 2025); a full list of the packages used can be found in Table S7. The significance threshold was set to 0.05 (Wei et al. 2006; Waldmann 2018), and results are reported to 3 decimal places and 1 for percentages.

### Ethical note

This study draws on a long-term dataset from an ongoing monitoring program of a GSL colony and was collected using both invasive and noninvasive methods. Although the original field protocols were not designed specifically for this project, the use of these data maximizes the scientific value of the effort put into the field work. As the study was conducted in the Galápagos Islands, all ethical oversight was provided by the local authorities. Field procedures were reviewed and authorized by the Galápagos National Park Directorate (DNPG) and followed the ethical guidelines set forth by Sikes and Gannon (2011) and the ASAB Ethical Committee (2025). When administering passive integrated transponder tags and external flipper-tags (Allflex ear tags size 0; Allflex, United Kingdom), the application site was disinfected with alcohol before and after tagging. Individuals with fore-flippers deemed too small (<9 cm across the wrist) were not tagged; instead, a shallow fur clipping was made on the rump for temporary identification. Animals that appeared weak or in poor condition were not captured to prevent additional stress. When intervention was appropriate (ie weaned individuals who remained on the islet), the DNPG was notified. Anesthesia was not used during captures due to associated risks; captured individuals were instead restrained within the hoop nets (Fuhrman Diversified Inc., Seabrook, TX, United States) used for capture. Captures occurred only under conditions that were safe for both the sea lion and field personnel, and only when the animal was positioned on low-risk substrate (eg sand or vegetation). During handling, care was taken to secure the individual’s head and to ensure that the surrounding area was free of hazards. Handling time was minimized as much as possible (∼15 min), and breathing rates were continuously monitored for signs of acute stress. All animals were observed immediately post-release for indicators of stress, injury, or behavioral changes and were monitored periodically throughout the remainder of the day. Animal welfare was prioritized throughout all stages of fieldwork, and protocols were reviewed annually to ensure alignment with current ethical and welfare standards.

## Results

### Change in pupping numbers

**Figure 3:**
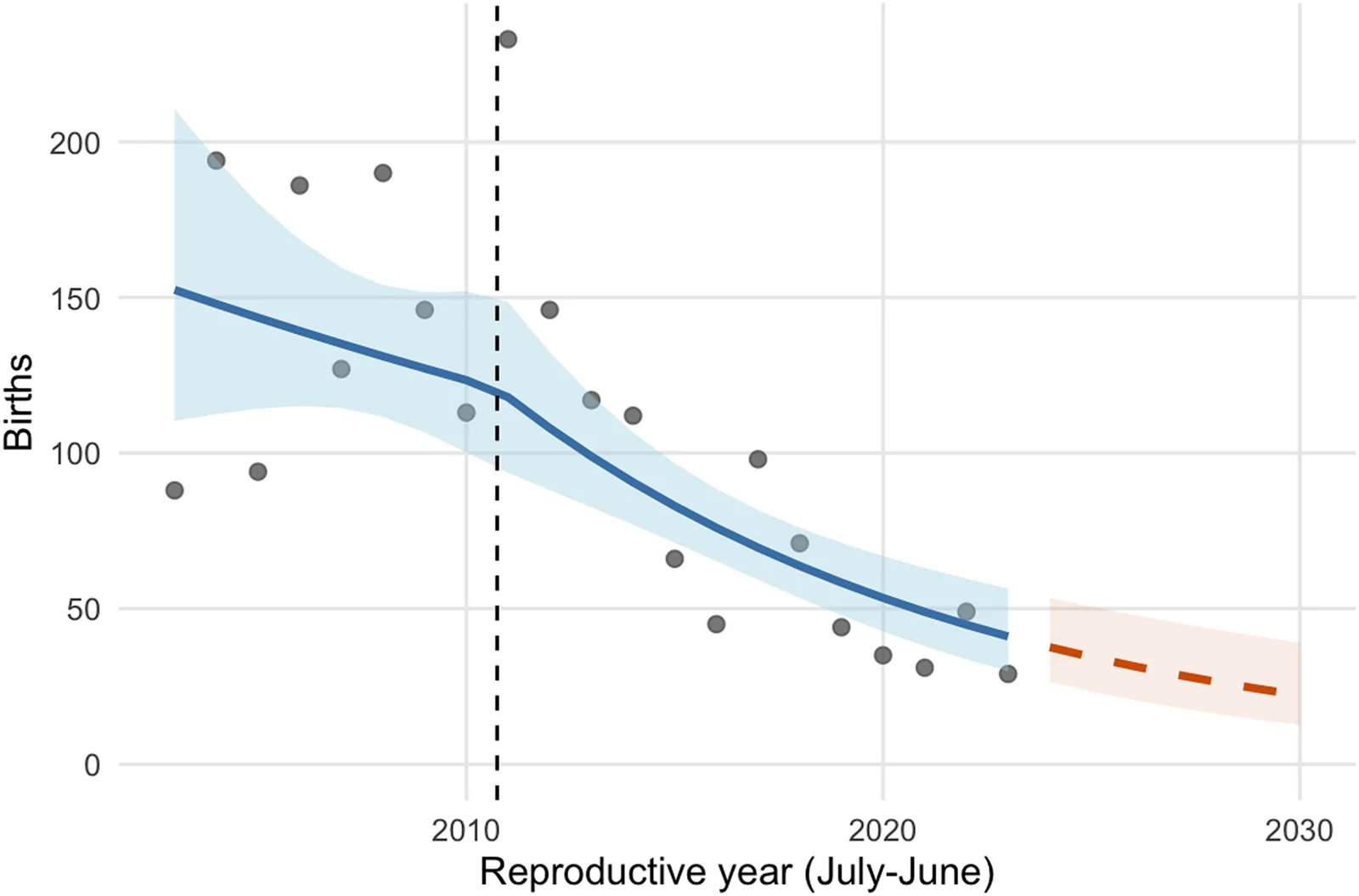
Birth rate from 2003 to 2023 (solid line), with the predicted rate until 2030 (dashed line). The shaded area around the birth rate is the 95% CI. The vertical dashed line represents the onset of significant decline in births (breakpoint = 2010.7 [March 2011, Gregorian calendar]), and the points represents the number of births recorded for each reproductive year. Birth counts were adjusted for annual effort via a log-offset in the model. The predicted birth rate was calculated at a constant reference effort equal to the mean annual effort (88 d).

Over the data collection period (2003 to 2023), 2,214 births were recorded in the colony, of which 1998 had known DoB (see Supplementary material 2.1). A mean of 105 ± 60 pups was born (95 ± 54, known DoB) per reproductive year with high interannual variation. When calculating these metrics using only years with both cold and hot field seasons—which were more likely to accurately capture extreme births—the annual mean number of births was 106 ± 61 (94 ± 53 known DoB), from a total of 1,803. A GAM confirmed that the decline began in approximately the March of reproductive year 2010. We found a positive correlation between effort and the number of births observed in a year (Spearman’s *ρ* = 0.842, *n* = 21, *P* < 0.001). Prior to the breakpoint, the birth rate showed a nonsignificant decline of 3.0% per year (Rate Ratio [RR] = 0.97 per year, 95% CI: 0.92 to 1.03, *P* = 0.317). In contrast, the postbreakpoint birth rate decreased significantly by ∼8.4% per year (RR = 0.92 per year, 95% CI: 0.88 to 0.95, *P* < 0.001), corresponding to a 58.5% decline per decade (a half-life ≈ 7.9 yr) (Fig. 3).

Female counts and births per year exhibited similar trends to the predicted birth rate (Fig. S2). Both the number of females and births decline significantly over the study period (*P* < 0.001) with no evidence that the rate of decline differed between the 2 groups (year × series interaction: *β* = −2.469 ± 1.76 standard error [SE], *P* = 0.168). We found no significant differences in the proportions of sexes born across the study period using a χ^2^ test (χ^2^ = 13.20, df = 20, *P* = 0.869 (Fig. S3).

**Figure 4:**
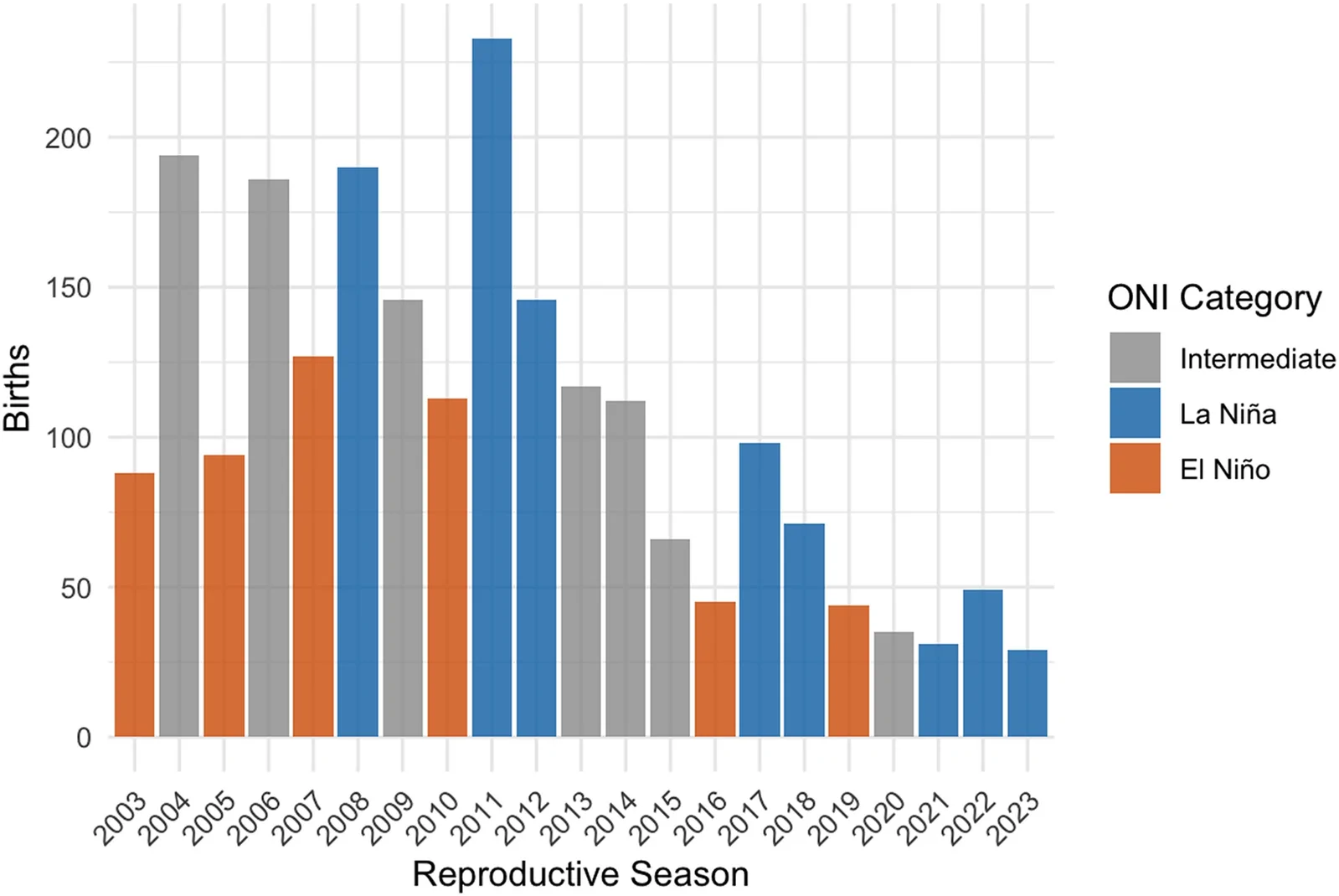
The number of births recorded every year during the study colored according to the ONI category for the first 6 mo of the (partum) year (range: 29-233).

We found no effect of any climate variable (ONI, anomaly and SST) at any scale (1 yr and 6 mo) for either the pre- or postpartum year, on the number of pups born in the colony (Fig. 4). We also found no evidence of any effect of colony density on the number of births (Table S2).

### Pupping period

**Figure 5:**
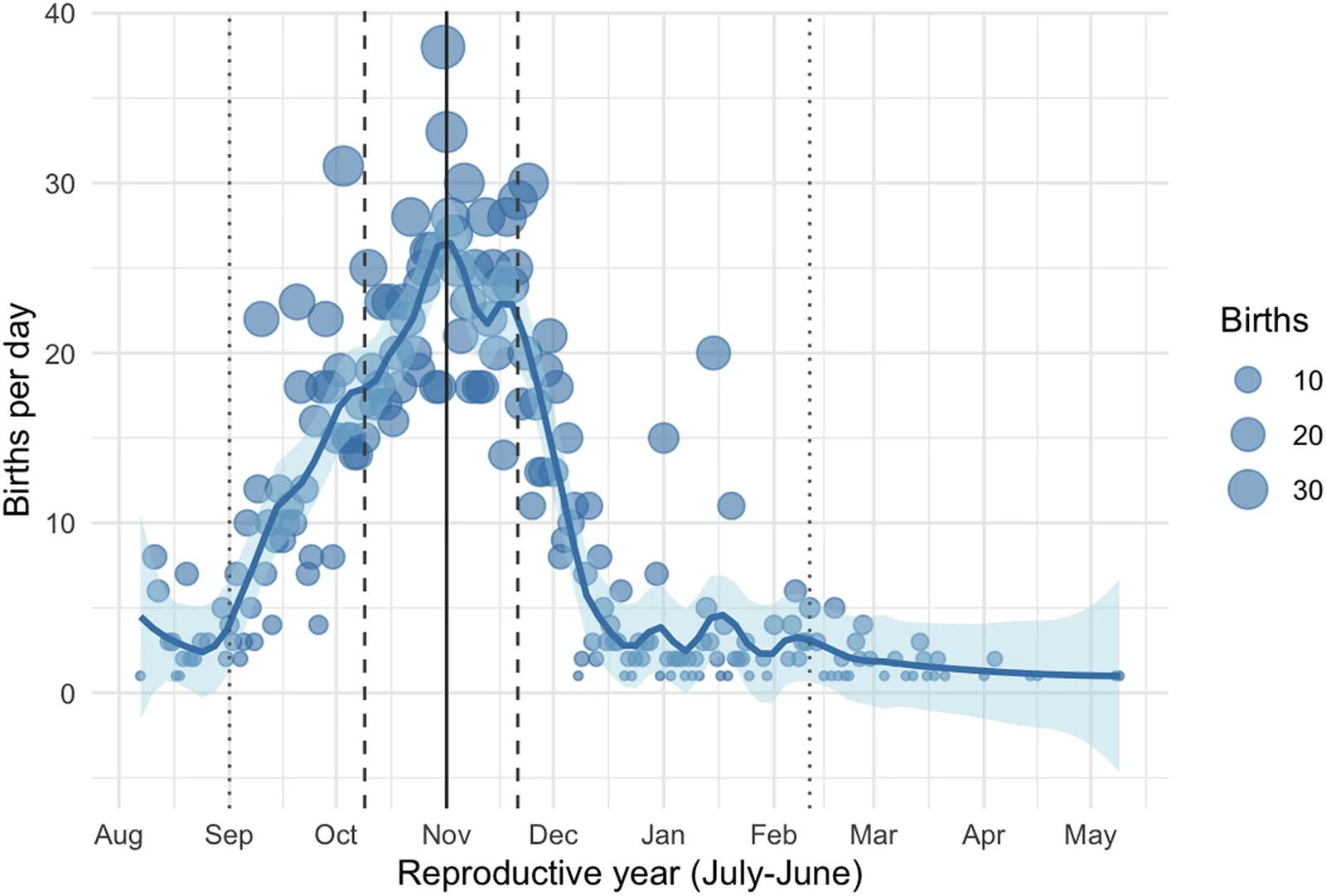
Distribution of all known births (*n* = 1,998) mapped onto a single reproductive year. Weighted dots represent the number of births registered to each day of the month; the solid line represents the trend in births and the shaded area surrounding it the 95% CI. Overall MPP is represented by the central vertical solid line (1 November), immediately on either side are the boundaries (dashed) of the 50% pupping window (9 October to 21 November), and further out the 95% pupping window (dotted) (1 September to 11 February).

The earliest known DoB recorded during the study period was 7 August and the latest 5 May, with 50% of all pups born between 9 October–21 November and 95% between 1 September–11 February (Fig. 5). The overall MPP of the study period was 1 November with a maximum difference of 47-d between the earliest and latest annual median recorded (34-d when excluding years that had no hot field season). An LM (model 1B, Table S3) found no temporal trend in MPP (*β* = 0.097 ± 0.42 SE, *P* = 0.026); however, the absence of a hot field season has a significant effect resulting in an MPP ie 27 ± 7 d earlier (*P* = 0.001, *R*^2^ = 0.542). We found no effect of the duration of the pupping period, climate, colony density, or maternal age (Table S3). LM model 3A_1_ did show that MPP is more sensitive to La Niña oscillations (partum year) that cause a nonsignificant shift of MPP earlier in the year (*β* = 9.145 ± 5.03 SE, *P* = 1.161, *R*^2^ = 0.576). However, this effect should be treated with caution since AICc favored the simpler model. When investigating the effect of maternal age, the results may have been influenced by the increasing mean age alongside the increasing proportion of mothers with a known age (Fig. S4).

**Figure 6:**
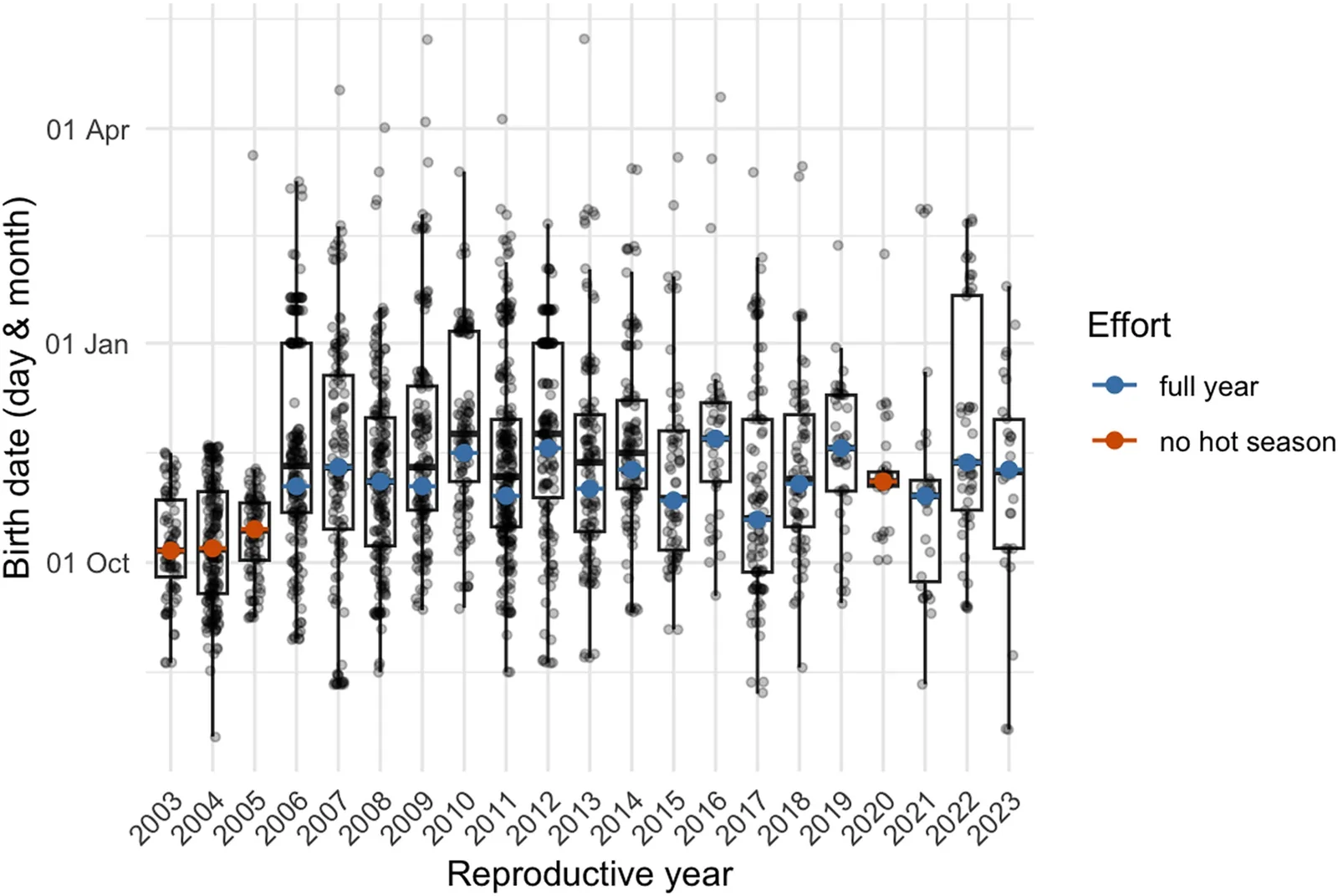
Distribution of births (points) for each reproductive year with interquartile range. Median pupping point for each year is indicated in color with blue (2006-2019, 2021-2023) representing years where there were 2 field seasons for data collection covering the reproductive period and immediately afterward (hot and cold) and red (2003-2005, 2020) for years with only 1 field season covering the reproductive period (cold). The exception is 2020 where data were only collected for a very brief period during the hot season (late April) due to COVID-19 restrictions.

The mean duration of the pupping period was 186 ± 49 d (range of 88 to 260 d, Fig. 6). When excluding years with no hot field season, these values changed to a mean of 201 ± 37 d (range of 118 to 260 d). The final selected model 6C (Table S4) showed a significant decline in pupping period duration over the study period (*β* = −6.010 ± 1.45 SE, *P* = 0.001, *R*^2^ = 0.445) although effort significantly shapes the data (*P* = 0.002). We also found that the mean number of juveniles present in the colony has a significant negative impact on the duration (*β* = −0.938 ± 0.28 SE, *P* = 0.004). We found no effect of breakpoint, number of births, MPP, climate, or average maternal age (Table S4).

When attempting to explain the large amount of variation in the timing of births within the pupping period, we found that pup DoB within a year varied significantly according to sex, the onset of colony decline (breakpoint), and ONI category of the reproductive year. Male pups are, on average, born earlier than females by nearly 5 d (*β* = −4.730 ± 1.68 SE, *P* = 0.005), and births after the breakpoint are later (*β* = 23.748 ± 7.38 SE, *P* = 0.005). The effect of ONI depended on the breakpoint. La Niña conditions were associated with later births prior to the onset of the decline (*β* = 19.316 ± 9.37 SE, *P* = 0.057), this effect was significantly reduced post-breakpoint (interaction *β* = −35.817 ± 12.06 SE, *P* = 0.009), resulting in earlier births during La Niña years post-breakpoint relative to intermediate conditions. El Niño oscillations showed no significant interaction with the timing of births (model 4A_3_, *n* = 1,982, Table S5). Pups with a higher birth SMI were born significantly earlier in the season (*β* = −2.594 ± 0.85 SE, *P* = 0.002) (model 6A, *n* = 1,528); however, we found no effect of maternal age at parturition. We found no effect of maternal foraging strategy on the timing of birth when including an interaction term with the mean SST of the 6 mo prior to the start of the reproductive year. However, when using the mean SST of the first 6 mo of the reproductive year as an interaction term, we found that pelagic foraging mothers gave birth significantly later than benthic foragers under mean SST conditions (*β* = 19.750 ± 7.12 SE, *P* = 0.007). There was a notable significant SST × strategy interaction (*β* = −18.965 ± 8.62 SE, *P* = 0.032), indicating that pelagic individuals exhibited a stronger advancement to earlier births under warmer SST conditions.

### Yearling survival

We found that on average 65.9% of all pups born into the colony survive to their first year. Early investigation found that sampling effort during an individual’s yearling year did not significantly affect the probability of being identified as having survived. These results guided our inclusion of individuals who were not observed as yearlings but were recorded on the islet when older (≥2 yr of age) (*n* = 94), and for whom no measurement of effort was available. We found significant effects of all climate variables where warmer conditions negatively affect survival, however mean anomaly during a pup’s first year of life, with reproductive year as a random intercept (model 1B, Table S6), showed the greatest improvement to the model and is negatively associated with survival (*β* = −0.463 ± 0.15 SE, *P* = 0.001, *n* = 2,185). The mean number of juveniles present in the colony (model 4E) during a pup’s first year had a significant positive effect on survival to yearling age (*β* = 0.006 ± 0.00, *P* = 0.015, *n* = 2,185), as did the timing of an individual’s birth (model 6A), where pup’s born later were more likely to be observed the following year (*β* = 0.002 ± 0.00 SE, *P* = 0.037, *n* = 2,185). However, in both cases, BIC did not show an improvement in the model (Table S9). MPP also showed an effect on survival but is likely an artifact of the data collection structure shaping the median. SMI had a significant positive effect on survival with each unit increase in body condition associated with a 52% increase in the odds of survival (*β* = 0.418 ± 0.15 SE, *P* > 0.001, *n* = 1,517). The range of SMI in our study was 3.244 to 10.783. We found no effect of breakpoint, sex, maternal age, or the number of same age conspecifics within an individual’s surrounding birth site, on survival probability (Table 1). When examining birth site, site 12 (Fig. 2) was used as the reference as yearling survival probability for that area was closest to that of the colony average (65.2%, 65.9%, respectively). We found that birth location affects the probability of pup survival; however, births were unevenly distributed, and sample sizes for many sites were very small preventing us from drawing any meaningful conclusion (model 7A, Table S9).

**Table 1:** The relationship of each of the selected predictors with pup survival probability to yearling age.

Model index	Parameter	*β*	SE	*P*	*nind*	BIC
0B	Breakpoint	−0.121	0.091	0.183	2,185	2,793.32
1B	Mean anomaly (climate)	−0.463	0.147	**0**.**002**	2,185	2,730.66
2A	Sex	0.055	0.094	0.560	2,169	2,704.73
3B	Effort	−0.001	0.001	0.321	2,141	2,675.78
4E	Mean juveniles (density)	0.007	0.003	**0**.**016**	2,185	2,733.29
5A	MPP	−0.016	0.007	**0**.**030**	2,185	2,734.32
6A	DoB	0.002	0.001	**0**.**037**	2,185	2,733.79
7A	Birth site	—	—	—	1,066	1,555.06
7B	Birth site pup density	0.002	0.006	0.786	1,066	1,330.15
8A	SMI	0.418	0.079	**>0.001**	1,517	1,922.32
9A	Maternal age	−0.060	0.049	0.223	273	324.01

For full model builds, parameters, and model selection, please refer to Table S6. Predictors with a significant relationship are highlighted in bold. No values can be provided for birth site as the predictor was tested as a factor; for full results of model 7A, please refer to Table S9.

## Discussion

We found that the Caamaño colony has been in decline since monitoring began in 2003 (−3% annually). However, in March of the 2010 reproductive year (March 2011 Gregorian calendar), a significant decline (−8.4% annually) in the number of births began, which cannot be attributed to climate effects or reduced female fecundity. Although overall MPP has not changed, since the 2010 reproductive year the duration of the pupping period has shortened significantly (∼6 d per year), ie not related to effort, and females have become more sensitive to La Niña events that directly impact the timing of births. We found that female niche choice (Trappes et al. 2022), in the form of foraging strategy, as well as offspring sex and birth condition (SMI) significantly affects the timing of parturition within a reproductive year. Although ENSO does affect pup survival to yearling age, there has been no temporal change in survival over the study period.

Poor environmental conditions are associated with a decline in pupping probability in GSL as well as other pinniped species, such as Australian fur seals (*Arctocephalus pusillus doriferus*) and gray seals (Gibbens and Arnould 2009; Smout et al. 2020; Páez-Rosas et al. 2021). However, neither climate nor colony density had a significant effect on the annual number of births within the Caamaño colony. The presence of colony decline since the inception of data collection, although unexpected, is likely a reflection of the overall population decline this species is experiencing (Riofrío-Lazo et al. 2017; Krüger and Trillmich 2026). Although we cannot fully eliminate the impact data collection may have had on the colony, we feel that it is unlikely to be a driving factor in the colonies decline since the significant acceleration from the 2010 reproductive year onward began a decade after the study’s start and predates the use of biologgers by several years. We therefore suggest that climate as a proxy for resource availability and its impact on pupping decline on this species can only be fully understood when both environmental and competition effects are accounted for.

As expected, the number of births declined in proportion to the number of females present in the colony. This, coupled with the increasing mean age of mothers and the rising proportion of known-age females, indicates that the decline in births is not driven by diminishing fecundity but rather reduced recruitment. The Caamaño colony is considered a closed population (Trillmich et al. 2016). Although females born outside the colony are occasionally recruited, throughout the entire study period there has been only a single confirmed sighting of a tagged individual outside of Caamaño or the closest mainland town, Puerto Ayora (∼1.5 km), the latter of which is not a breeding site. Coupled with the high site fidelity observed in this species (Wolf and Trillmich 2007), it is unlikely that individuals born in the studied colony are recruiting elsewhere in numbers large enough to cause the observed population decline. Rather, reduced recruitment to the colony may be a result of increased mortality at the juvenile stage before individuals reach breeding age. A similar pattern has been documented in Steller sea lions (*Eumetopias jubatus*, Holmes and York 2003) and California sea lions (*Zalophus californianus*) where for the latter peak annual survival (males and females) is only reached at age 5 (DeLong et al. 2017).

We found no overall trend in MPP; however, the observed tendency toward earlier MPP during La Niña conditions suggests that higher resource abundance could be contributing to gestating females reaching full term earlier. This relationship with resource availability follows previous findings in South American sea lions (*O. flavescens*, Soto et al. 2004). That MPP’s positive relationship with La Niña becomes significant after 2010 is likely due to the subsequent decrease of breeding females in the colony and resulting reduction in individual variation represented in the data, rather than a change in maternal pupping behavior. The timing of the pupping period, and therefore MPP, is likely constrained by females’ dependence on seasonal increases in resource availability caused by the cold (Garúa) season (Palacios 2004; Jeglinski et al. 2015); any significant shift in reproductive timing would therefore be dependent on permanent seasonal changes.

Model fits indicated that the effects of climate on MPP and pupping period duration are weak; however, this tentative relationship may be due to the general cohort-specific way climate means were calculated, masking more acute individual sensitivity to ENSO. Despite this, our findings still support previous work that has highlighted the negative impact of poor resource availability on maternal reproductive success (Páez-Rosas et al. 2021; Schwarz et al. 2022) and pup SMI at birth (Mueller et al. 2011) in GSL. Fetal development has been associated with maternal size, age, and reproductive history in northern fur seals (*Callorhinus ursinus*, Trites 1991). Although this may also be true of GSL, it is unlikely that fetal growth, in both mass and length, is independent of maternal resource availability, and further investigation at an individual level is needed in this area. Gestation duration and developmental aging have been shown to be remarkably consistent across individuals in recent ultrasound assessments of California sea lions (Fiorucci et al. 2024). This suggests that environmental variability may have a stronger influence on neonate condition than on the absolute timing of parturition. We found that pups born earlier in the season had a higher SMI, and males were born 5 d earlier than females on average. Gestational differences in development and duration according to offspring sex have been observed in other mammals (Nogalski and Piwczyński 2012; Broere-Brown et al. 2016; Meakin et al. 2021). However, given the prolonged duration of the pupping period in GSL, this potential sex effect cannot fully explain the high amount of variation observed in the timing of births. Variation in parturition is therefore more likely to arise through condition-dependent processes, such as differences in implantation timing (Boyd 1984; Reijnders et al. 2010) or carry-over effects from reproductive attempts in the previous year (Dezeure et al. 2022). The carry-over effect could have been particularly acute in our study given the aging structure of the female population. This may also explain the significant negative effect of juvenile presence on pupping period duration, with more females potentially taking a 2-yr average birth interval contributing to a reduction in pup births. As the number of breeding females decreases, their pupping behavior may inadvertently align to maximize mutual benefits, reducing the pupping period duration despite their asynchronous breeding cycle. The extended lactation characteristic of GSL, among the longest of any pinniped, together with the frequent occurrence of supersuckling behavior (Childs et al. 2026a, 2026b) may further exacerbate this pattern by prolonging maternal investment and increasing the likelihood of extended birth intervals and a shorter pupping period duration.

Reproductive mechanisms are shaped by fluctuations in resource availability and, at the individual level, differences in foraging proficiency and reproductive history (Páez-Rosas et al. 2021; Geeson et al. 2022; Schwarz et al. 2022). Mothers may also exhibit adaptive changes in maternal investment during lactation to buffer offspring against interannual climate variations (Mueller et al. 2011; Kraus et al. 2013). We found that pelagic foraging females appear to demonstrate such flexibility or sensitivity to SST variability through shifts in birth timing. Earlier parturition under warmer conditions may represent a compensatory response, potentially aligning peak lactation demands with more favorable foraging windows or reducing energetic constraints of pregnancy during tough foraging conditions (as observed in *Capreolus capreolus,* Kauffert et al. 2025). At a species-wide level, interisland variation in parturition timing may be dictated by local bathymetry influencing species-specific prey availability and therefore individual foraging strategy specialization within a colony (Villegas-Amtmann et al. 2009; Páez-Rosas and Aurioles-Gamboa 2014; Jeglinski et al. 2015; Schwarz et al. 2021). We would therefore expect any trends associated with climatic variation to differ across the archipelago due to local specialization. Such flexibility could partially buffer offspring against climate variability or protect the female’s long-term reproductive condition. However, despite this apparent adjustment, a continued decline in our study population’s recruitment suggests that these compensatory mechanisms may be insufficient under sustained warming. This pattern may indicate that Caamaño’s population is approaching or has surpassed a demographic tipping point (de Silva and Leimgruber 2019), limiting its capacity for recovery (Hutchings 2015).

Our results found no indication that the probability of pup survival to yearling age was affected by the onset of significant population decline towards the end of the 2010 reproductive year. This further supports our suggestion that the root of the pupping decline is associated with changes in survival at the juvenile stage prior to reproductive maturity. It also implies that maternal buffering behavior during offspring early life is independent of population density. However, female GSL adaptability does not fully buffer climatic variability, with warmer oceanographic temperatures during a pup’s first year reducing survival probability. It is likely that this is the result of reduced maternal foraging success, increased maternal expenditure and/or lower quality, and reduced material transference (Mueller et al. 2011; Jeglinski et al. 2015; Eddy et al. 2019). The importance of early-life maternal investment as a key determinant of fitness was further highlighted by the positive relationship between earlier births and higher SMI, with each unit increase in SMI associated with a 52% increase in the odds of surviving to yearling age. This is consistent with previous work on this species (Mueller et al. 2011) and suggests that climatic influences may operate, at least in part, through the mother.

The mean number of juveniles present in the colony during a pup’s first year was positively associated with survival. Although model selection criteria suggest that this effect should be interpreted with caution, it does draw attention to current gaps in our understanding of the social lives of this cooperative, philopatric, and highly tactile species. Increased juvenile presence may reflect favorable environmental conditions or provide social or behavioral benefits, such as enhanced social cohesion, increased vigilance against predators, and improved motor skill development that contribute to increase survival probability (Laroche et al. 2008; Heintz et al. 2017; Nagel et al. 2022). Reduced conspecific density in northern elephant seals (*Mirounga angustirostris*) is negatively associated with weaning mass (Holser et al. 2021), and social grouping is a successful Cape fur seal strategy against shark predation (*Arctocephalus pusillus pusillus*, Laroche et al. 2008). Strong social bonds have also been linked to fitness and success of allied male bottlenose dolphins (Gerber et al. 2022), and the inheritance of social bonds from mothers has been shown to increase longevity in offspring in other social species (Stanton and Mann 2012; Evans et al. 2021; Ilany et al. 2021). Although we may not be able to fully explain the effect of juvenile presence on early pup survival in this population, it raises questions about the importance of social interactions in a declining colonial mammal, consistent with concerns highlighted in previous work on this species (Zenth et al. 2021).

### Future directions

This study highlights that future work with GSLs should move beyond treating population dynamics as simple linear trends, as this approach risks masking critical demographic events both within and among colonies. Failure to account for intercolony variation may obscure early warning signs of decline, particularly in smaller colonies that are inherently more vulnerable to negative pressures, as suggested by census data (Riofrío-Lazo et al. 2017). The focus colony of this study, once among the largest and healthiest in Santa Cruz, now appears to be functionally reduced with only a single tagged individual observed outside of Caamaño and Puerto Ayora (personal observations), highlighting the need for finer-scale temporal and spatial analyses. A pronounced and unexplained population decline from March 2011 (Gregorian calendar) suggests the occurrence of a major stressor that cannot be readily attributed to documented climate drivers and may instead reflect anthropogenic impacts. This decline predates the deployment of biologging technologies on the colony by several years, limiting our ability to retrospectively assess behavior and habitat use during this critical period. The apparent lack of recruitment of younger mothers further points to reduced survival to breeding age, for which data are currently lacking in this species. Potential mechanisms include increased predation pressure (Lucas and Stobo 2000; Schwarz et al. 2013), competition for resources at shared foraging sites, such as seamounts, frequented by local fisheries (Keledjian and Mesnick 2013), increasing tourism—shown to significantly affect population health in other pinnipeds (Cowling et al. 2014; Back et al. 2018; Suraci et al. 2021), and unrecorded human–wildlife conflict, including fisheries interactions and bycatch, which are known threats across pinniped species worldwide (Keledjian and Mesnick 2013; Denkinger et al. 2015; Sepúlveda et al. 2023). Addressing these knowledge gaps will require integrative approaches combining long-term demographic monitoring, juvenile-focused studies, and explicit consideration of anthropogenic pressures.

This work underscores the critical importance of incorporating data from smaller, often-overlooked satellite colonies into broader assessments of population trends. By documenting cumulative ecological changes at a remote island site, our study reveals how localized dynamics may diverge from those observed at larger, more accessible monitoring sites. The long-term dataset presented here provides a rare and timely opportunity to emphasize the value of remote-site monitoring, which should be included in a coordinated regional census effort focused on island-based sea lion populations. Our findings advocate for a more inclusive and spatially distributed approach to pinniped island population monitoring—one that recognizes the ecological importance of isolated colonies, and how demographic changes in these sites may drive broader site interconnectedness and distribution within this highly social species.

## Conclusion

Our results confirm that GSLs are highly adapted to the extreme variability of their climate and that their continued decline is likely driven by decreased juvenile survival resulting in reduced recruitment at primiparity. The adaptation of conservation strategies to ensure the recovery of this species requires further research into understanding survival between weaning age and primiparity. If future work shows that anthropogenic effects are associated with population decline, then arguably these effects are easier to reverse and robust conservation strategies more attainable at the local level.

## Supplementary Material

arag107_Supplementary_Data

## Data Availability

Analyses reported in this article can be reproduced using the data provided by Childs et al. (2026c).

## Inclusion and diversity statement

Our study is composed of a mixed gender team. All field work included scientists based in the country where the study was conducted, and who are residents of the local area in which the study was performed.
