# Supplementary material for: Maternal flexibility in Galápagos sea lions mitigates climate impacts but not population decline
Source: Behav Ecol. 2026 Sep 1;37(6):arag107. doi: 10.1093/beheco/arag107 (PMC13598343; doi:10.1093/beheco/arag107)
Supplement: Supplementary file 1 [file arag107_supplementary_data.pdf]

Maternal flexibility in Galápagos Sea Lions mitigates climate impacts but not population decline

Maternal flexibility in Galápagos Sea Lions

Behavioural Ecology

Authors (*ORCiD number*)**:** Childs, Alexandra K. (0009-0003-4150-6745) *†[1]; Stainfield, Claire[2] (0009-0009-6190-4058); Stoehr, Svenja[1] (0009-0008-0287-2140); Twiss, Sean D.[3] (0000-0002-1923-8874); Krüger, Oliver[1,4] (0000-0002-8066-4936).

***Corresponding Author:** Alexandra K. Childs; achilds17@gmail.com

Supplementary materials:

2. Methods

2.1 *Data collection*

Date of birth (DoB)

All marked individuals were assigned a date of birth (DoB) which was categorised according to their accuracy where: 'exact' indicates that the birth was observed directly or the pup was found a few hours post-birth, 'weak' an estimate accurate to within ± 2 weeks, and 'unknown' was a researcher estimation ranging from 3 weeks to multiple months/years. Throughout the rest of the study the 'exact' and 'weak' categorisations are referred to as 'known' births. An additional category, 'rough' (± a field season or approximately 6 months), was created for individuals with an 'unknown' DoB who were handled for the first time at under 6 months of age as these estimates have been shown to be highly accurate (Jeglinski et al. 2010). 'Rough' DoB were used to better understand the number of pups born each year but were removed for any analyses that investigated effects related to the timing of births or duration of the pupping period.

2.2 *Data treatment*

Birth-site and birth-site pup-density

The location of all observed births was recorded according to the subdivision of the islet shown in Figure 2, however mothers sometimes move their pups to a different site shortly after birth. During the first month of life pups are highly site faithful due to their still developing mobility, we therefore took birth-site to be the site in which the pup was most frequently observed during their first month of life. This reclassification accounted for pups who were born close to site borders and may have wandered into the neighbouring site at a very young age, and for mothers who happened to birth in a compromised location and relocated their pup very soon after birth. We then used the reclassified birth-site to calculate the birth-site pup-density which we took to be the sum of the total number of births of the focal site and all sites with which it shares a border. We decided on this inclusive total as pups tend to creche together once they become more mobile and social cohesion appears to be site dependent (*personal observation*). This inclusive approach allowed us to account for any social benefits presented by higher conspecific density.

Effort

When investigating the timing of births and duration of the pupping period, effort was calculated in three different forms to reflect variation in data collection. For analyses of birth timing, effort was defined as the total number of observation days per year. However, when investigating the duration of the pupping period this measure was less appropriate due to the inclusion of “weak” births (i.e. births occurring between cold and hot field seasons). Instead, it was deemed more appropriate to measure effort as the window of observation effort, calculated as the number of days (inclusive) between the very first and last observation round of a reproductive year. This measure more accurately captured how much of the pupping period

was observed in each year. To further account for variation in sampling structure, a second measure of effort was included as a binary variable indicating whether both a cold and hot field season were present (“hot field season”). This allowed earlier years (2003–2005), which lacked a hot field season, to be retained in the analysis while accounting for differences in data collection design. Data collection over two field seasons (particularly hot seasons) were more likely to identify births at the extremes of the pupping period with reasonable accuracy (± 2 weeks). In contrast, the first three cold field seasons of the study (2003-2005) were also longer, so that effort calculated as the number of observation days was not directly comparable to later years with split sampling periods.

A third measure of effort, defined as the total number of observation rounds per year (2–5 per day), was also explored during preliminary analyses. However, this metric was not retained, as it showed weaker relationships with response variables than the number of observation days (see SM R Markdown and HTML output).

El Niño Southern Oscillation

The El Niño Southern Oscillation (ENSO) is a seasonal climate pattern that consists of stochastic environmental events El Niño and La Niña. El Niño events are characterised by high sea surface temperatures (SST) and low oceanic productivity which results in poor food resources for the Galápagos Sea Lion (GSL). In contrast La Niña events are associated with low SST and high productivity creating resource abundance for GSL (Trillmich et al. 2014; NOAA 2023). Oceanic Niño Index (ONI) is the rolling 3-month average temperature anomaly used to classify the strength and occurrence of ENSO events. Values of +0.5 or higher indicate El Niño and values of -0.5 or lower indicate La Niña. All values that fall in-between these are classed as ‘Intermediate’ and are characterised by relatively stable environmental conditions.

For every year we calculated 4 mean values using monthly anomaly and daily local SST values (Figure S1). Each of these mean values was assumed to broadly represent different biological phases of maternal investment in their pup, spanning either annual or 6-month temporal scales:

- The first 6-months (July-December) of the partum year represented early maternal investment over a period of exponential offspring growth (Mueller et al. 2011).
- The partum year (July-June) represented maternal investment in a pup that is nutritionally dependent on its mother, and her ability to navigate the local annual variations in climate and therefore resource availability as dictated by the ‘hot’ and ‘cold’ (Garúa) seasons (see Figure 1).
- The last 6-months (January-June) of the pre-partum year represented maternal investment during late gestation and/or her capacity to carry her offspring to term (Mueller et al. 2011).
- The pre-partum year (July-June) represented fertilisation and maternal gestational investment as well as a mother’s ability to navigate the local annual variations in climate and therefore resource availability (‘hot’ and ‘cold’(Garúa) seasons [Figure 1]) over that period.

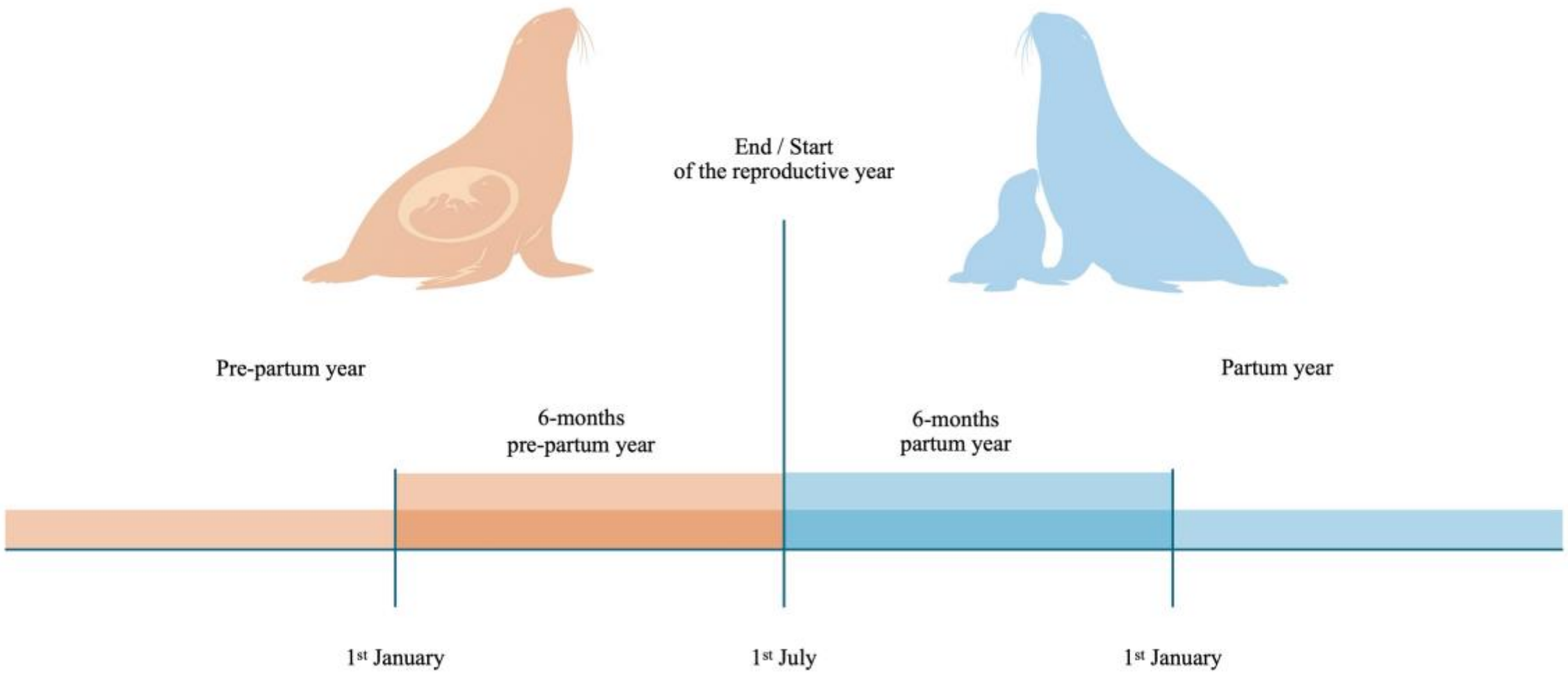

Figure S1. The breakdown of all climate variables into annual means of the pre- and partum year, and 6-month means of the first 6-months of the partum year and last 6-months of the pre-partum year. Start date of the reproductive year was taken to be 1st July, analogously to Childs et al. (2026), to capture extremely early 'rough' births at the end of July.

Mean anomaly values, and subsequently ONI categories, were used as a proxy for resources available to mothers (Trillmich & Limberger 1985; Soto et al. 2004). Previous exploratory work into this species behaviour has indicated that the effects of ENSO are sometimes clearer and easier to interpret when mean anomaly values are broadly classified into the ONI categories. It is for this reason that when investigating climate effects both forms of the ENSO (anomaly & ONI category) were tested.

2.3 *Statistical Analysis*

Pupping duration

Prior to the construction of the linear models (LM) used for assessing effects related to temporal changes in the duration of the pupping period (reported in the main text). A series of preliminary LMs were used to test the impact of effort on results. We used the primary dataset covering the study period and a subset that began in 2006 after the implementation of a hot field season. The year 2020 was excluded from all data sets due to COVID19 restrictions on fieldwork. We fitted

LMs with pupping duration as the response variable. Two models were compared for each dataset: a simple model including only the year to test for temporal trends, and an extended model including both year and sampling effort (effort window) to assess whether effort explained variation in pupping period duration. This approach allowed us to distinguish between biological trends and potential artefacts arising from differences in observation window length. Model performance was evaluated using statistical significance of predictors, model fit and AICc.

Sea Surface Temperature

Sea surface temperature (SST) has been shown to explain foraging success when included as an interaction term with maternal foraging strategy (Schwarz et al. 2022). SST was prepared in the same manner as ENSO anomaly (3 values per year) and was tested in all models as a proxy for local productivity during early exploration. However, in all cases when significant relationships with the dependent variable were found ONI and/or anomaly had a greater significant relationship and/or showed a greater improvement to the model. We have therefore not reported the use of SST during analysis or any results associated with it, with the exception of its use as an interaction effect with maternal foraging strategy. Results of models including SST values can be found in the supplementary .html output and .Rmd file.

Climate effects

Table S1. A breakdown of all climate and colony parameters during hypothesis testing where 'X' indicates a variables inclusion in the model with *brkpt* (breakpoint) included as a fixed effect and/or an interaction effect. It is important to note that colony density variable *Total* excludes pup counts, and *All adults* includes unidentified individuals of an androgenous appearance.

Parameter class	Parameter	Colony pupping health (NB GLM)		Pupping period						Yearling survival (LRM)	
				Median pupping point (LM)		Pupping period duration (LM)		Pupping timing (LMM)			
		brkpt **included**	***brkpt*** **as interaction**	***brkpt*** **included**	***brkpt*** **as interaction**	***brkpt*** **included**	***brkpt*** **as interaction**	***brkpt*** **included**	***brkpt*** **as interaction**	***brkpt*** **included**	***brkpt*** **as interaction**
ONI category	Prepartum year	X	X	X	X	X		X	X		
	Prepartum 6 months (Jan –June)	X	X	X	X	X					
	Partum year	X	X	X	X	X		X	X	X	X
	Partum 6 months (July – Dec)	X	X	X	X	X					
Anomaly	Prepartum year	X	X	X	X	X		X	X		
	Prepartum 6 months (Jan –June)	X	X	X	X	X	X				
	Partum year	X	X	X	X	X		X	X	X	X
	Partum 6 months (July – Dec)	X	X	X	X	X					
SST	Prepartum year	X	X	X	X	X		X	X		
	Prepartum 6 months (Jan –June)	X	X	X	X	X					
	Partum year	X	X	X	X	X		X	X	X	X
	Partum 6 months (July – Dec)	X	X	X	X	X					
Colony density	Total, annual mean	X	X	X	X	X		X	X	X	
	Males, annual mean	X	X	X	X	X		X	X	X	
	Females, annual mean	X	X	X	X	X		X	X	X	
	Females, annual maximum	X	X	X	X	X		X	X		
	All adults, annual mean	X	X	X	X	X		X	X	X	
	Juveniles, annual mean	X	X	X	X	X	X	X	X	X	X
	Total births			X	X	X		X	X		

Change in pupping numbers

During early analysis two Spearman's rank correlation tests were used to individually determine the effect of the number of observation days, and number of identification rounds conducted during a reproductive year on the number of births recorded. The results informed us that effort was most appropriately represented by the number of observation days per year.

The GAM to investigate the onset of pupping decline was specified with a Poisson error distribution, a log link function and fitted using restricted maximum likelihood estimation (Wood 2011). We initially set a minimum threshold of 3 consecutive negative points and confirmed the reliability of the result with a more conservative minimum threshold of 5.

To ensure numerical stability and prevent bias when modelling the number of births as both a rate per day and per female (adjusted for effort), zero values of effort and females were replaced with a minimal positive constant (1×10^{-6}) prior to log-transformation.

When examining the potential effects of climate and colony density the variables listed in Table S1 were tested individually within the preceding selected NB GLM as independent effects and an interaction effect with breakpoint. Only models that showed the greatest improvement to the model explanatory power for each climate measurement (anomaly, ONI, SST) are included in Table S2, for full model results and fits please refer to the original .Rmd and .html output provided in the online supplementary materials. Model 1A was selected as the final model (Table S2).

Table S2. All fitted NB GLMs predicting the number of pups born in a reproductive year as described by the predictors. The number of parameters (np) and number of years (nyr) for which the corresponding variables were available are used as a basis for comparison via the AICc. Variable notation *cohort* refers to the reproductive year, *brkpt* to the breakpoint as identified by a GAM model, $effort_{days,t}$ to the number of unique days during which births could have been recorded during reproductive year, $ONI_{delayed,t}$ to the ONI category for the year prior to a reproductive year, $anom_{6mo,t}$ the mean anomaly for the first 6 months of a reproductive year, $SST_{6mo,t}$ the mean SST for the first 6 months of a reproductive year, $\overline{N}_{nonpup,t}$ to the mean number of individuals observed in the colony – excluding pups – during a reproductive year, $\overline{N}_{adultM,t}$ the mean number of adult males observed in the colony during a reproductive year, $\overline{N}_{adultF,t}$ the mean number of adult females observed in the colony during a reproductive year, $\overline{N}_{juv,t}$ the mean number of juveniles (1-5 years of age) observed in the colony during a reproductive year. The index in bold indicates the model that was preferred at each stage of model selection, model 1A was selected as the final model.

Index	predictors	offset	AICc	nyr	np
0A	-	-	*231.89*	21	1
0B	*cohort*	-	*215.33*	21	2
**0C**	*cohort, brkpt*	-	***214.47***	21	3
**1A**	*cohort, brkpt*	$effort_{days,t}$	***195.28***	20	3
1B	*cohort, brkpt*	-	*206.77*	20	3
$2A_{B3}$	*cohort, brkpt* * $ONI_{delayed,t}$	$effort_{days,t}$	*207.47*	20	7
$2B_{C3}$	*cohort, brkpt* * $anom_{6mo,t}$	$effort_{days,t}$	*199.15*	20	5
$2C_{C3}$	*cohort, brkpt* * $SST_{6mo,t}$	$effort_{days,t}$	*200.99*	20	5
$3B_1$	*cohort, brkpt,* $\overline{N}_{nonpup,t}$	$effort_{days,t}$	*198.84*	20	4
$3K_1$	*cohort, brkpt,* $\overline{N}_{adultM,t}$, $\overline{N}_{adultF,t}$, $\overline{N}_{juv,t}$	$effort_{days,t}$	*206.79*	20	6

2.2. Pupping period

To explore whether changes in the within-year distribution of births could be associated with variation in the duration of the pupping period, we calculated a log-transformed asymmetry index describing the spread of births around the median date for each year and used a one-sample *t*-test to evaluate whether mean asymmetry differed from zero. A Wilcoxon rank-sum test was used to assess whether birth spread asymmetry differed between hot and non-hot field seasons.

Table S3. All fitted LMs predicting the median pupping point (MPP) of a reproductive year as described by the predictors. The number of parameters (np) and number of years (nyr) for which the corresponding variables were available are used as a basis for comparison via the AICc and informed by adjR^2. Variable notation *cohort* refers to the reproductive year, *births* to the total number of births recorded that reproductive year, *brkpt* to the breakpoint as identified by a GAM model, $effort_{days,t}$ and $effort_{season,t}$ to the number of unique days during which data was collected and whether a hot field season was conducted that reproductive year, respectively, $duration_{,t}$ to the duration in days of the pupping period, $ONI_{,t}$ to the ONI category for the reproductive year, $N_{maxF,t}$ to the maximum number of females observed during a reproductive season, $\overline{m}_{age,t}$ to the age of the mother at birth,. The index in bold indicates the model that was preferred at each stage of model selection.

Index	predictors	AICc	adjR^2	nyr	np
0A	-	*170.92*	*0.000*	21	1
**0B**	*cohort*	***168.03***	***0.195***	21	2
0C	*cohort*, $births_{,t}$	*170.54*	*0.173*	21	3
0D	*cohort, brkpt*	*170.99*	*0.156*	21	3
1A	*cohort*, $effort_{days,t}$	*160.36*	*0.308*	20	3
**1B**	*cohort*, $effort_{season,t}$	***152.10***	***0.542***	20	3
1C	*cohort*	*161.20*	0.202	20	2
2	*cohort*, $effort_{season,t}$, $duration_{,t}$	*154.14*	*0.551*	20	4
$3A_1$	*cohort*, $effort_{season,t}$, $ONI_{,t}$ * *brkpt*	*156.63*	*0.771*	20	8
4	*cohort*, $effort_{season,t}$, $N_{maxF,t}$	*152.89*	*0.578*	20	4
5A	*cohort*, $effort_{season,t}$, $\overline{m}_{age,t}$	*136.79*	*-0.166*	17	4
**5B**	*cohort*, $effort_{season,t}$	***133.51***	***-0.138***	17	3

Table S4. All fitted LMs predicting the duration of the pupping period of a reproductive year as described by the predictors. Reproductive year 2020 was removed due to the high levels of missing information. The number of parameters (np) and number of years (nyr) for which the corresponding variables were available are used as a basis for comparison via the AICc and informed by adjR^2. Variable notation *cohort* refers to the reproductive year, *births* to the total number of births recorded that reproductive year, $effort_{season,t}$ to whether a hot field season was conducted that reproductive year, *brkpt* to the breakpoint as identified by a GAM model, $MMP_{,t}$ to the median pupping point of the reproductive year, $ANOM_{,6mo\text{-}partum}$ to the mean anomaly for the first 6 months of the partum reproductive year (proxy for early maternal investment in a live pup), $N_{juv,t}$ to the mean number of juveniles observed during a reproductive season, $\overline{m}_{age,t}$ to the age of the mother at birth. The index in bold indicates the model that was preferred at each stage of model selection.

Index	predictors	AICc	adjR^2	nyr	np
**0A**	-	***215.21***	***0.000***	20	1
0B	*cohort*	*218.00*	*-0.055*	20	2
1A	$effort_{season,t,}$	*209.98*	*0.293*	20	2
**1B**	*cohort*, $effort_{season,t,}$	***207.17***	***0.445***	20	3
2	*cohort*, $effort_{season,t,}$, *brkpt*	*209.86*	*0.437*	20	4
3	*cohort*, $effort_{season,t,}$, $births_{,t}$	*210.75*	*0.412*	20	4
4	*cohort*, $effort_{season,t,}$, $MPP_{,t}$	*209.21*	*0.455*	20	4
$5B_4$	*cohort*, $effort_{season,t}$, $ANOM_{,6mo\text{-}partum}$	*207.89*	*0.490*	20	4
**6C**	*cohort*, $effort_{season,t}$, $N_{juv,t}$	***200.27***	***0.652***	20	4
7A	*cohort*, $effort_{season,t}$, $N_{juv,t}$, $\overline{m}_{age,t}$	*163.62*	*0.460*	16	5
**7B**	*cohort*, $effort_{season,t}$, $N_{juv,t}$,	***161.68***	***0.420***	16	4

Table S5. All fitted LMMs predicting the timing of a pup's birth within the reproductive period as described by the predictors and random effect included. The number of parameters (np) and number of individuals (nind) for which the corresponding variables were available are used as a basis for comparison via the BIC. Variable notation $effort_{days,t}$ and $effort_{rounds,t}$ refers to the number of unique observation days and total number of identification rounds over the course of a reproductive year when a birth could have been observed, *brkpt* to the breakpoint as identified by a GAM model, *sex* to pup sex, $ONI_{,t}$ to the overall ONI category for a pup's year of birth, $SST_{delayed,t}$ to the mean SST for the year prior to a pup's year of birth, $\overline{N}_{juveniles,t}$ to the mean number of juveniles in the colony on a given year, *SMI* to the SMI of a pup before 10 days, m_{age} to the age of the mother at parity, $SST_{6mo,t}$ to the mean SST for the first 6 months of a pup's year of birth, $SST_{delayed6mo,t}$ to the mean SST for the 6 months immediately prior to a pup's year of birth. Index refers to the steps in model selection. The index in bold indicates the model that was preferred at each stage of model selection.

Index	random effect	predictors	BIC	nind	np
0A		-	*20354.05*	1998	1
**0B**	*cohort*	-	***20191.49***	1998	2
1A	*cohort*	$effort_{days,t}$	*19809.30*	1962	3
**1C**	*cohort*	-	***19806.28***	1962	2
**2**	*cohort*	*brkpt*	***20189.93***	1998	3
**3**	*cohort*	*sex, brkpt*	***20028.57***	1982	4
**$4A_3$**	*cohort*	*sex, brkpt* * $ONI_{,t}$	***20026.09***	1982	8
$4C_6$	*cohort*	*sex, brkpt* * $SST_{delayed,t}$	*20026.57*	1982	6
4D	*cohort*	*sex, brkpt* * $ONI_{,t}$, *brkpt* * $SST_{delayed,t}$	*20026.92*	1982	10
$5C_1$	*cohort*	*sex, brkpt,* $\overline{N}_{juveniles,t}$	*20034.26*	1982	9
**6A**	*cohort*	*sex, brkpt* * $ONI_{,t}$, *SMI*	***14181.43***	1528	9
6B	*cohort*	*sex, brkpt* * $ONI_{,t}$	*14184.88*	1528	8
7A	*cohort*	*sex, brkpt* * $ONI_{,t}$, m_{age}	*2727.47*	271	9
**7B**	*cohort*	*sex, brkpt* * $ONI_{,t}$	***2726.91***	271	8
**8A**	*cohort*	*sex, brkpt* * $ONI_{,t}$, m_{for} * $SST_{6mo,t}$	***749.96***	79	11
**8B**	*cohort*	*sex, brkpt* * $ONI_{,t}$, m_{for} * $SST_{delayed6mo,t}$	***740.45***	79	11
8C	*cohort*	*sex, brkpt* * $ONI_{,t}$	*787.92*	79	8

2.3. Yearling survival

Table S6. All fitted LRMs predicting the probability of a pup to survive to yearling age described by the predictors and random effect included. The number of parameters (np) and number of individuals (nind) for which the corresponding variables were available are used as a basis for comparison via the BIC. Variable notation $anom_{,t}$ refers to the mean anomaly during an individual's first year of life, *brkpt* to the breakpoint as identified by a GAM model, *sex* to pup sex, $effort_{days,t}$ and $effort_{rounds,t}$ to the number of unique observation days and total number of identification rounds when a pup could have been observed as a 1-year-old, $\overline{N}_{juveniles,t}$ to the mean number of juveniles (1-5years) in the colony during a pup's first year, $median_{,t}$ to the median pupping point for an pup's year of birth, *DoB* to a pups Julian day of birth, $birth_{site}$ to the location a pup was born in, $birth_{density}$ to the number of same-age conspecifics found within and around a pup's place of birth, *SMI* to the SMI of a pup before 10 days, m_{age} to the age of the mother at parity. Index refers to the steps in model selection. The index in bold indicates the model that was preferred at each stage of model selection.

**Index**	**random effect**	**predictors**	**BIC**	**nind**	**np**
0A		-	*2787.40*	2185	1
0B		*brkpt*	*2793.32*	2185	2
**0D$_3$**		$anom_{,t}$ * *brkpt*	***2762.34***	2185	2
1A	*cohort*	$anom_{,t}$ * *brkpt*	*2742.17*	2185	5
**1B**	*cohort*	$anom_{,t}$	***2730.66***	2185	3
1C	*cohort*	-	*2731.15*	2185	2
2A	*cohort*	$anom_{,t}$, *sex*	*2704.73*	2169	4
**2B**	*cohort*	$anom_{,t}$	***2697.38***	2169	3
3A	*cohort*	$anom_{,t}$, $Effort_{days,t}$	*2676.53*	2141	4
3B	*cohort*	$anom_{,t}$, $Effort_{rounds,t}$	*2675.78*	2141	4
**3C**	*cohort*	$anom_{,t}$	***2669.07***	2141	3
4E	*cohort*	$anom_{,t}$, $\overline{N}_{juveniles,t}$	*2733.29*	2185	4
5A	*cohort*	$anom_{,t}$, $median_{,t}$	*2734.32*	2185	4
6A	*cohort*	$anom_{,t}$, *DoB*	*2733.79*	2185	4

7A	*cohort*	$anom_{,t}$, $birth_{site}$	*1555.06*	1066	47
7B	*cohort*	$anom_{,t}$, $birth_{density}$	*1330.15*	1066	4
**7C**	*cohort*	$anom_{,t}$	***1323.26***	1066	3
**8A**	*cohort*	$anom_{,t}$, *SMI*	***1922.32***	1517	4
8B	*cohort*	$anom_{,t}$	*1944.68*	1517	3
9A	*cohort*	$anom_{,t}$, m_{age}	*324.01*	273	4
**9B**	*cohort*	$anom_{,t}$	***319.84***	273	3

Table S7. All packages used during data manipulation and analysis.

Package	Authors
MuMln (Multi-Model Inference)	Bartoń 2025
lme4 (Linear mixed-effects models)	Bates, Mächler, Bolker, & Walker 2015
Matrix	Bates, Maechler & Jagan 2025
lubridate	Grolemund & Wickham 2011
lmerTest	Kuznetsova, Brockhoff, & Christensen 2017
emmeans	Lenth & Piaskowski 2025
report	Makowski et al. 2023
nlme	Pinheiro, Bates & R Core Team 2025
Mixed-Effects Models in S and S PLUS (book)	Pinheiro & Bates 2000
R (base environment)	R Core Team 2025
broom	Robinson et al. 2025
Modern Applied Statistics with S	Venables & Ripley (2002)
ggplot2	Wickham 2016
dplyr	Wickham et al. 2023
purrr	Wickham & Henry 2025
scales	Wickham, Pedersen, & Seidel 2025
tidyr	Wickham, Vaughan & Girlich 2024
mgcv / GAM references	Wood 2003, 2004, 2011, 2016, 2017
knitr	Xie 2014, 2015, 2025
zoo	Zeileis & Grothendieck 2005
Car / R News diagnostic article	Zeileis & Hothorn 2002
kableExtra	Zhu 2024

3. Results

3.1. Change in pupping numbers

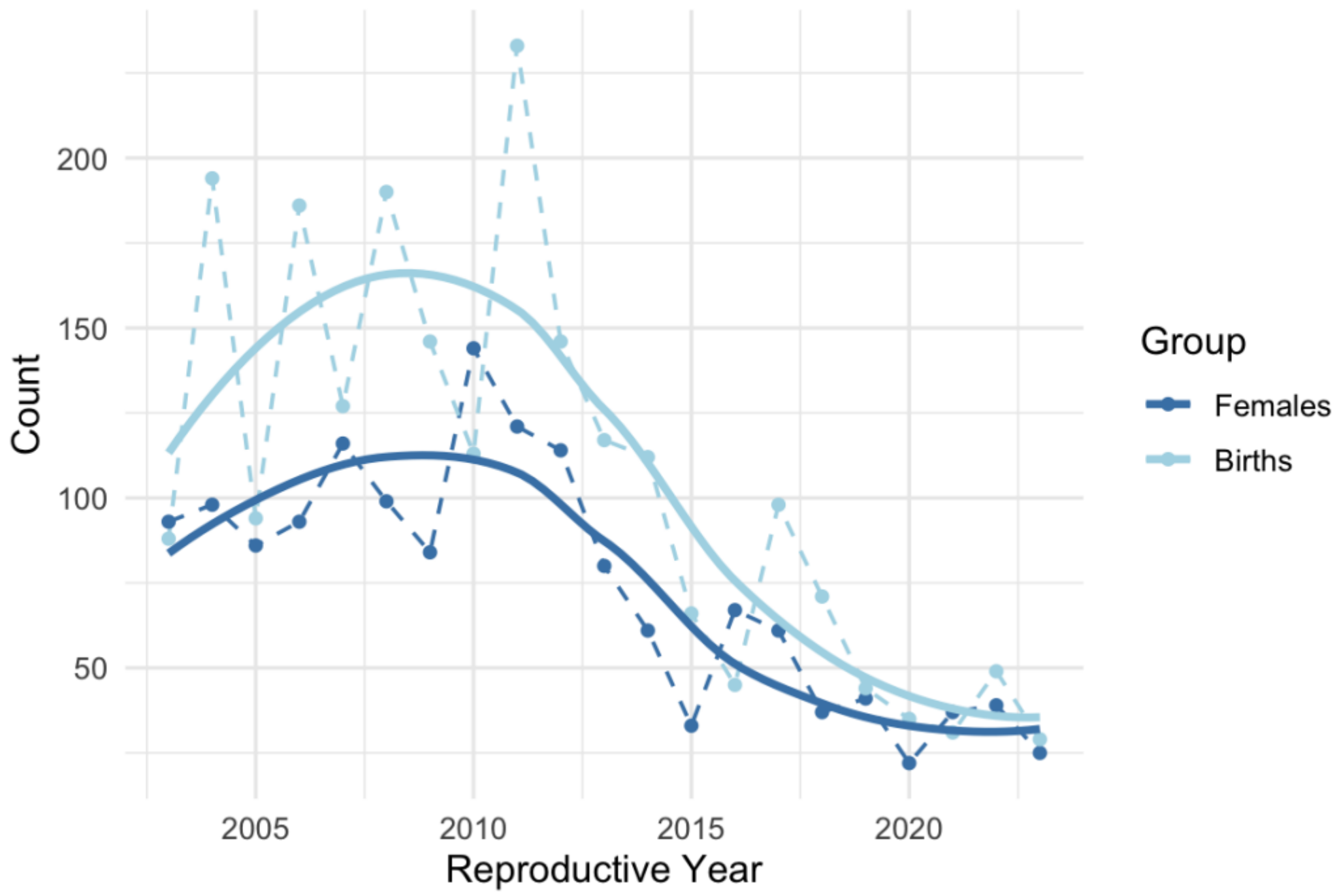

Figure S2. The number of births (pale blue) and maximum number of females (dark blue) observed each year (points). Smoothed lines (solid) show the trends of each group over the study period (2003-2023).

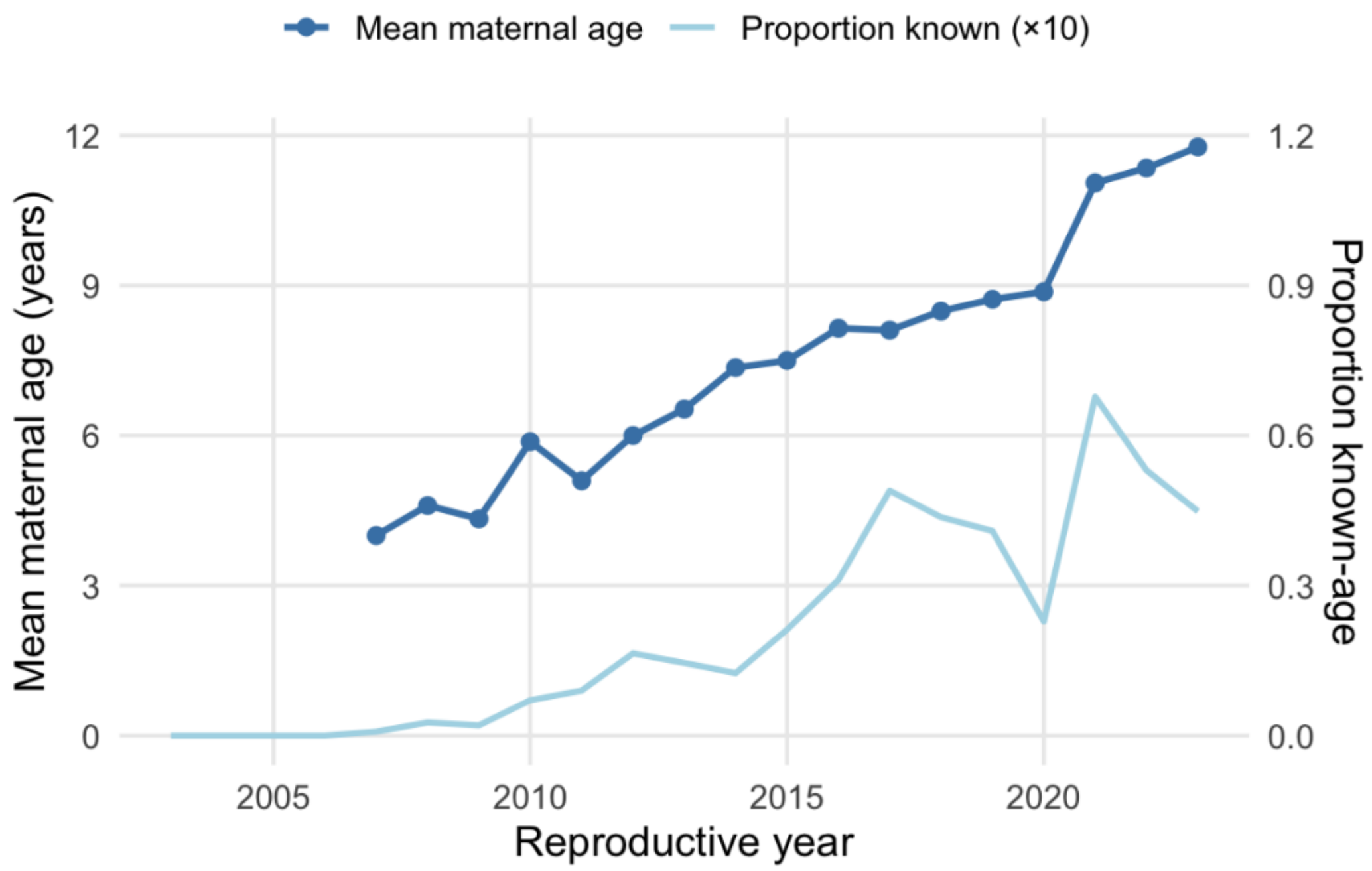

Figure S4. Average age of mothers with a known DoB who gave birth (dark blue), and the proportion of all mothers with a known DoB (pale blue), per reproductive year.

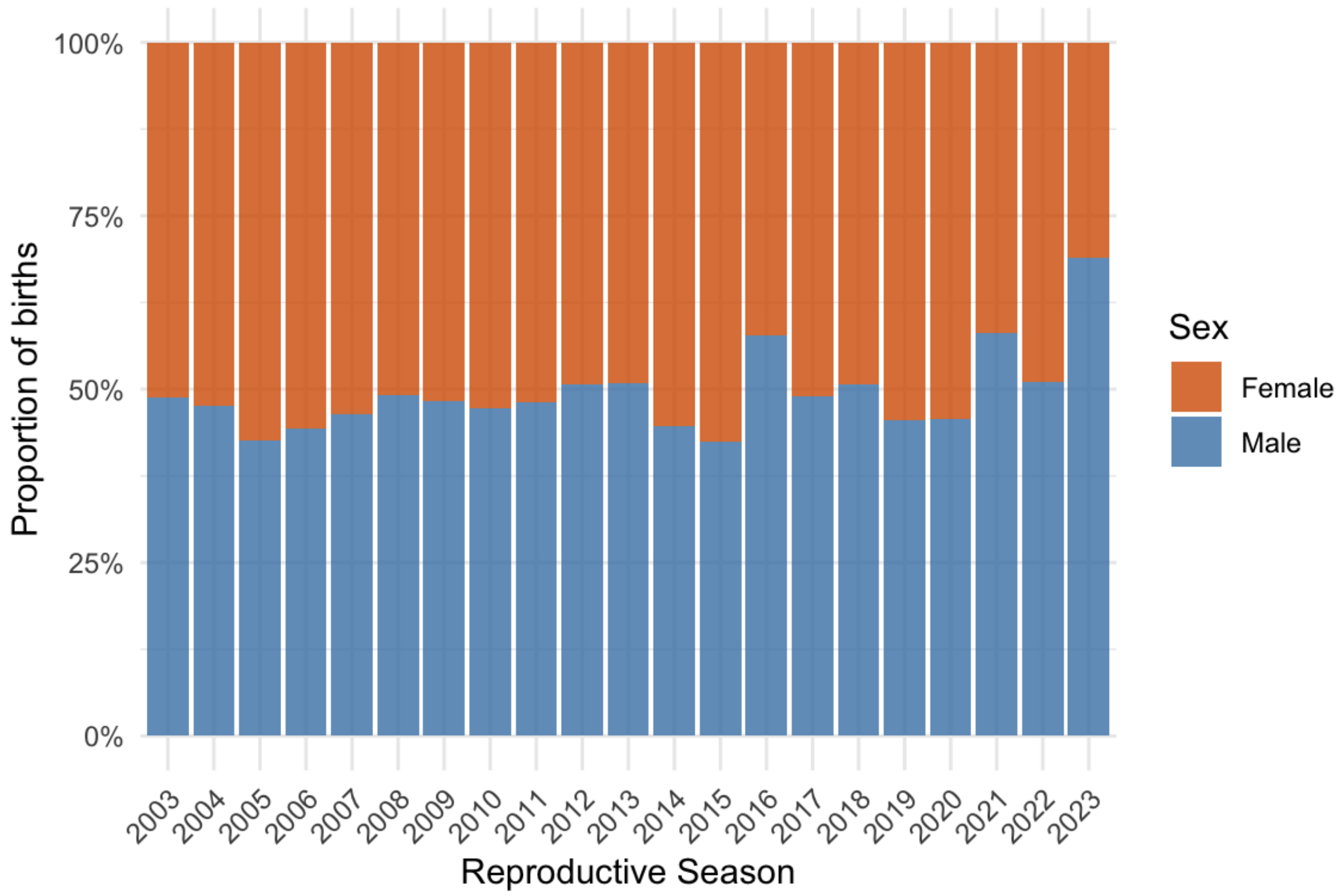

Figure S3. Proportion of male (blue) and female (red) pups born every year during the study period.

Exact births

During the study period there were 1543 births directly observed with an average of 77 pups born per year (± 48). When excluding years with no data collection during a 'hot' season these metrics changed to a total of 1252 births and 74 (± 49) per annum. A GAM model identified the onset of the decline in births (breakpoint) occurred in 2010.3 (approximately October 2010 [reproductive year]). Effort had a significant effect on the number of births observed (Spearman's $\rho = 0.645$, $p = 0.002$). Prior to the breakpoint, the birth rate showed a non-significant decline of 3.9% per year (RR = 0.96 per year, 95% CI: 0.91–1.02, $p = 0.176$). Post-decline there is a near-significant decrease in the birth rate by approximately 10.5% per year (RR = 0.90, 95% CI: 0.86–0.93, $p < 0.092$), corresponding to a 67.1% decline per decade.

Female counts and births per year exhibited similar trends to the birth rate. Both the number of females and births decline significantly over the study period ($p < 0.001$) with no evidence that the rate of decline differed between the two groups (year x series interaction: $\beta = -1.495 \pm 1.53$ *SE*, $p = 0.335$). We found no significant differences in the proportions of sexes born across the study period using a Chi-squared test ($\chi^2 = 45.621$, $df = 19$, $p = 0.682$). We found that mean anomaly of the first 6-months of the partum year has a significant negative effect on the number of births with each unit increase in anomaly reducing the birth rate by approximately 11% ($\beta = -0.118 \pm 0.05$ *SE*, $p = 0.021$). We found no evidence of any effect of colony density on the number of births.

3.2. Pupping period

When including all years pupping duration showed no temporal trend ($\beta = -0.782 \pm 1.58$ *SE*, $p = 0.626$) but was positively associated with sampling effort ($\beta = 0.501 \pm 0.17$ *SE*, $p = 0.010$, $R^2 = 0.255$), indicating that longer observation periods resulted in longer estimated durations. However, when excluding early years that did not have a hot field season (2003-2005), pupping duration declined significantly over time ($\beta = -4.077 \pm 1.72$ *SE*, $p = 0.033$), while sampling effort had no significant effect ($\beta = 0.065 \pm 0.238$ *SE*, $p = 0.789$, $R^2 = 0.299$) and did not improve model fit. These results indicate that the apparent influence of effort in the full dataset is driven by early years with reduced sampling, and that, once these are excluded, the observed decline in pupping duration reflects a genuine temporal trend rather than an artefact of variation in sampling effort.

When calculating the asymmetry of the spread of births around the median for each year we found no evidence that mean asymmetry differed from zero ($p = 0.081$). Asymmetry also did not differ significantly between years which included or excluded data collected during a hot

field season (p = 0.635), although years including a hot field season showed a larger mean birth spread (23–31.2 days) compared to years without (11–15.2 days).

All climate and colony density variables were tested individually both as independent effects and interacting with the breakpoint (Table S1). For full results of each of these models please see the original .Rmd and .html output provided in the online supplementary materials.

Exact births

The earliest DoB recorded during the study period was August 18th and the latest 4th April, with 50% of all known births occurring between 14th October – 19th November, and 95% between 13th September – 30th December. The overall median pupping point was 1st November, with a 35-day difference between the earliest and latest median recorded (14 days when excluding years with no 'hot' field season). We found no temporal trend in MPP (model 1B, $\beta = 0.245 \pm 0.39$ *SE*, $p = 0.537$) however the absence of a hot field season has a significant effect resulting in an MPP that is 22 ± 7 days earlier ($p = 0.003$, $R^2 = 0.526$). We found no effect of the duration of the pupping period, climate, colony density of any sex and age class or maternal age (Table S3). LM model $3A_2$ did show that MPP is more sensitive to La Niña oscillations (partum year, with breakpoint included as an interaction term) that cause a significant shift of MPP to later in the year after the breakpoint ($\beta = -22.649 \pm 8.68$ *SE*, $p = 0.023$, $R^2 = 0.643$). However, this effect should be treated with caution since AICc favoured the simpler model. When investigating the effect of maternal age, the results may have been influenced by the increasing mean age alongside the increasing proportion of mothers with a known age (Figure S4).

The mean duration of the pupping period was 100 days ± 44 days (range of 36 - 207 days). When years with no hot field season were removed these metrics changed to a mean duration of 107 days ± 43 days (range of 36 – 207 days). Contrary to results when including births that were not directly observed, we found that the number of births had a significant positive effect on pupping period duration ($\beta = 0.628 \pm 0.24$ *SE*, $p = 0.021$) and improved the model, whereas the juvenile effect vanished. We found no effect of breakpoint, MPP, climate, density or average maternal age. The final selected model (guided by AICc) was in this case model 3: duration ~ cohort + effort + births (AICc 203.83, $n = 20$), which showed no temporal decline ($\beta = 0.293 \pm 2.37$ *SE*, $p = 0.903$).

When investigating the large amount of variation in the timing of births within the pupping period, we found that pup DoB within a season varied significantly according to the onset of the colony decline (breakpoint), and ONI category of the reproductive year. Births after the breakpoint are later ($\beta = 14.716 \pm 6.35$ *SE*, $p = 0.036$) and the effect of ONI depended on the breakpoint. La Niña conditions were associated with later births prior to the breakpoint ($\beta = 19.386 \pm 8.05$ *SE*, $p = 0.031$). After the breakpoint births under La Niña conditions are significantly earlier ($\beta = -26.212 \pm 10.74$ *SE*, $p = 0.028$), showing a complete reversal of the relationship. El Niño oscillations showed no significant interaction with the timing of births (model $4A_3$, $n = 1543$). Pups with a higher birth SMI were born significantly earlier in the season ($\beta = -2.088 \pm 0.85$ *SE*, $p = 0.014$) (model 6A, $n = 1421$). We found no effect of maternal foraging strategy on the timing of birth when including an interaction term with the mean SST of the 6 months prior to the start of the reproductive year (model 8B, $n = 51$). However, when using the mean SST of the first six months of the reproductive year as an interaction term (model 8A, $n = 51$), we found that pelagic foraging mothers gave birth significantly later than benthic foragers under mean SST conditions ($\beta = 12.264 \pm 6.87$ *SE*, $p = 0.048$). We found no

effect of effort, sex – likely due to many females being born after the cold field season was complete – density or maternal age.

3.3. Yearling survival

Table S8. Proportion of yearling survivors, along with the total number of births for each reproductive year included in this study. ONI category was derived from the mean annual anomaly of the reproductive year (i.e. year of birth).

Reproductive year	*n* births	Proportion of yearling survivors (%)	Mean anomaly	ONI category
2003	88	79.5	0.259	Intermediate
2004	194	70.6	0.543	El Niño
2005	94	85.1	-0.401	Intermediate
2006	186	68.8	0.272	Intermediate
2007	127	70.1	-1.165	La Niña
2008	190	53.2	-0.393	Intermediate
2009	146	56.8	0.743	El Niño
2010	113	76.1	-1.183	La Niña
2011	233	76.0	-0.673	La Niña
2012	146	66.4	-0.093	Intermediate
2013	117	54.7	-0.183	Intermediate
2014	112	57.1	0.525	El Niño
2015	66	33.3	1.740	El Niño
2016	45	82.2	-0.266	Intermediate
2017	98	74.5	-0.506	La Niña
2018	71	63.4	0.596	El Niño
2019	44	56.8	0.263	Intermediate
2020	35	85.7	-0.816	La Niña
2021	31	58.1	-0.849	La Niña
2022	49	65.3	-0.446	Intermediate
2023	29	NA	NA	NA

Table S9. The effect of pup birth site on the probability of survival to yearling age, where '*n* births' is the number of pup births observed in each site (see main text Figure 2) and '*n* yearlings' is the number of pups who survived from each site. Estimates (*β*) and *p* values were taken from model 7A (Table S6) with site '12' set as the reference level. Sites with a significant effect are marked in bold.

**Site ID**	***n* births**	***n* yearlings**	**Yearling survival (%)**	***β***	***p***
1	15	6	40.0	-1.816	0.694
2	31	19	61.3	-0.362	0.717
3	67	51	76.1	1.406	0.160
4	23	17	73.9	0.948	0.343
5	8	4	50.0	-0.750	0.453
6	1	1	100.0	0.004	0.997
7	4	4	100.0	0.007	0.994
8	10	8	80.0	0.897	0.370
9	2	2	100.0	0.005	0.996
10	16	14	87.5	1.563	0.118
11	181	123	68.0	1.057	0.290
12	155	101	65.2	-	-
13	44	34	77.3	1.670	0.095
14	14	11	78.6	1.019	0.308
15	18	14	77.8	1.050	0.294
16	21	14	66.7	0.243	0.808
**17**	**55**	**44**	**80.0**	**2.022**	**0.043**
18	14	10	71.4	0.185	0.853
19	10	7	70.0	0.252	0.801
20	7	4	57.1	-0.633	0.527
21	33	21	63.6	0.025	0.980
**22**	**43**	**36**	**83.7**	**2.103**	**0.035**
23	2	2	100.0	0.005	0.996
25	6	5	83.3	0.718	0.473
26	3	3	100.0	0.006	0.995
27	15	7	46.7	-1.496	0.135
28	149	96	64.4	-0.159	0.874
29	47	32	68.1	0.581	0.561
31	2	1	50.0	-0.553	0.581
32	1	0	0.0	-0.004	0.997
33	1	1	100.0	0.003	0.997

34	2	2	100.0	0.006	0.995
35	3	2	66.7	-0.185	0.854
36	8	6	75.0	0.502	0.616
m1b	1	0	0.0	-0.005	0.996
m2b	2	0	0.0	-0.004	0.997
m3b	2	1	50.0	-0.358	0.720
bb1b	2	1	50.0	-0.864	0.388
ca	7	1	14.3	-1.744	0.081
g1	4	3	75.0	1.268	0.205
peb	13	4	30.8	-1.642	0.101
pbb	1	0	0.0	-0.006	0.995
c1	4	1	25.0	-1.389	0.165
c2	8	6	75.0	0.984	0.325
**c3**	**11**	**3**	**27.3**	**-2.220**	**0.026**

Exact births

Over the entire study period 61.6% of all pups whose birth was directly observed survived to yearling age (n = 1543). We found significant effects of all climate variables where warmer conditions negatively affect survival. Mean anomaly during a pup's first year of life with reproductive year as a random intercept (model 1B) showed the greatest improvement to the model and is negatively associated with survival (β = -0.465 ± 0.16 *SE*, p = 0.005, n = 1530) however BIC favoured the simpler model. We found a significant positive effect of the mean number of adult females on pup survival (model 4C, β = 0.013 ± 0.00 *SE*, p = 0.018, n = 1530) but no juvenile effect, BIC favoured the simpler model. SMI had a significant positive effect on survival (model 7A, β = 0.404 ± 0.08 *SE*, $p < 0.001$, n = 1408). We found no effect of breakpoint, sex, effort, density, MPP, DoB, birth location, number of same age conspecifics or maternal age.

Bibliography

Bartoń, K. (2025) *MuMIn: Multi-model inference* (version 1.48.11) [R package]. Comprehensive R Archive Network (CRAN). https://doi.org/10.32614/CRAN.package.MuMIn

Bates, D., Mächler, M., Bolker, B. & Walker, S. (2015) Fitting linear mixed-effects models using lme4. *Journal of Statistical Software*, **67**, 1–48. https://doi.org/10.18637/jss.v067.i01

Bates, D., Maechler, M. & Jagan, M. (2025) *Matrix: Sparse and dense matrix classes and methods* (version 1.7-3) [R package]. CRAN. https://doi.org/10.32614/CRAN.package.Matrix

Childs, A.K., Feldmann, C., Stoehr, S., Demarthon, R.L., Piedrahita, P., Twiss, S.D. & Krüger, O. (2026) Milking it: repeated post-weaning suckling events in Galápagos sea lions (*Zalophus wollebaeki*). *The American Naturalist*. https://doi.org/10.1086/738773

Grolemund, G. & Wickham, H. (2011) Dates and times made easy with lubridate. *Journal of Statistical Software*, **40**, 1–25. https://www.jstatsoft.org/v40/i03/

Jeglinski, J.W.E., Mueller, B., Pörschmann, U. & Trillmich, F. (2010) Field-based age estimation of juvenile Galápagos sea lions (*Zalophus wollebaeki*) using morphometric measurements. *Aquatic Mammals*, **36**, 262–269. https://doi.org/10.1578/AM.36.3.2010.262

Kuznetsova, A., Brockhoff, P.B. & Christensen, R.H.B. (2017) lmerTest package: tests in linear mixed effects models. *Journal of Statistical Software*, **82**, 1–26. https://doi.org/10.18637/jss.v082.i13

Lenth, R. & Piaskowski, J. (2025) *emmeans: Estimated marginal means, aka least-squares means* (version 2.0.1) [R package]. CRAN. https://doi.org/10.32614/CRAN.package.emmeans

Makowski, D., Lüdecke, D., Patil, I., Thériault, R., Ben-Shachar, M. & Wiernik, B. (2023) Automated results reporting as a practical tool to improve reproducibility and methodological best practices adoption. Comprehensive R Archive Network (CRAN). https://easystats.github.io/report/

Mueller, B., Pörschmann, U., Wolf, J.B.W. & Trillmich, F. (2011) Growth under uncertainty: the influence of marine variability on early development of Galapagos sea lions. *Marine Mammal Science*, **27**, 350–365. https://doi.org/10.1111/j.1748-7692.2010.00404.x

NOAA Climate Prediction Center (2024) Oceanic Niño Index (ONI) data [online]. Available at: https://www.cpc.ncep.noaa.gov/data/indices/oni.ascii.txt (Accessed 20 June 2025).

Pinheiro, J., Bates, D. & R Core Team (2025) *nlme: Linear and nonlinear mixed effects models* (version 3.1-168) [R package]. CRAN. https://doi.org/10.32614/CRAN.package.nlme

Pinheiro, J.C. & Bates, D.M. (2000) *Mixed-effects models in S and S-PLUS.* Springer. https://doi.org/10.1007/b98882

R Core Team (2025) *R: a language and environment for statistical computing* [computer software]. R Foundation for Statistical Computing. Available at: https://www.R-project.org/

Robinson, D., Hayes, A. & Couch, S. (2025) *broom: Convert statistical objects into tidy tibbles* (version 1.0.8) [R package]. CRAN. https://doi.org/10.32614/CRAN.package.broom

Schwarz, J.F.L., DeRango, E.J., Zenth, F., Kalberer, S., Hoffman, J.I., Mews, S., Piedrahita, P., Trillmich, F., Páez-Rosas, D., Thiboult, A. & Krüger, O. (2022) A stable foraging polymorphism buffers Galápagos sea lions against environmental change. *Current Biology*, **32**, 1623–1628.e3. https://doi.org/10.1016/j.cub.2022.02.007

Soto, K.H., Trites, A.W. & Arias-Schreiber, M. (2004) The effects of prey availability on pup mortality and the timing of birth of South American sea lions (*Otaria flavescens*) in Peru. *Journal of Zoology*, **264**, 419–428. https://doi.org/10.1017/S0952836904005965

Trillmich, F. & Limberger, D. (1985) Drastic effects of El Niño on Galápagos pinnipeds. *Oecologia*, **67**, 19–22. https://doi.org/10.1007/BF00378445

Venables, W.N. & Ripley, B.D. (2002) *Modern applied statistics with S*. 4th edn. Springer. Available at: https://www.stats.ox.ac.uk/pub/MASS4/

Wickham, H. (2016) *ggplot2: Elegant graphics for data analysis*. Springer. Available at: https://ggplot2.tidyverse.org

Wickham, H., François, R., Henry, L., Müller, K. & Vaughan, D. (2023) *dplyr: A grammar of data manipulation*(version 1.1.4) [R package]. CRAN. https://doi.org/10.32614/CRAN.package.dplyr

Wickham, H. & Henry, L. (2025) *purrr: Functional programming tools* (version 1.1.0) [R package]. CRAN. https://doi.org/10.32614/CRAN.package.purrr

Wickham, H., Pedersen, T. & Seidel, D. (2025) *scales: Scale functions for visualization* (version 1.4.0) [R package]. CRAN. https://doi.org/10.32614/CRAN.package.scales

Wickham, H., Vaughan, D. & Girlich, M. (2024) *tidyr: Tidy messy data* (version 1.3.1) [R package]. CRAN. https://doi.org/10.32614/CRAN.package.tidyr

Wood, S.N. (2003) Thin-plate regression splines. *Journal of the Royal Statistical Society: Series B (Statistical Methodology)*, **65**, 95–114. https://doi.org/10.1111/1467-9868.00374

Wood, S.N. (2004) Stable and efficient multiple smoothing parameter estimation for generalized additive models. *Journal of the American Statistical Association*, **99**, 673–686. https://doi.org/10.1198/016214504000000980

Wood, S.N. (2011) Fast stable restricted maximum likelihood and marginal likelihood estimation of semiparametric generalized linear models. *Journal of the Royal Statistical Society: Series B (Statistical Methodology)*, **73**, 3–36. https://doi.org/10.1111/j.1467-9868.2010.00749.x

Wood, S.N. (2017) *Generalized additive models: an introduction with R*. 2nd edn. Chapman & Hall/CRC.

Wood, S.N., Pya, N. & Säfken, B. (2016) Smoothing parameter and model selection for general smooth models (with discussion). *Journal of the American Statistical Association*, **111**, 1548–1575. https://doi.org/10.1080/01621459.2016.1180986

Xie, Y. (2014) knitr: a comprehensive tool for reproducible research in R. In: Stodden, V., Leisch, F. & Peng, R.D. (eds) *Implementing reproducible computational research*. Chapman & Hall/CRC.

Xie, Y. (2015) *Dynamic documents with R and knitr*. 2nd edn. Chapman & Hall/CRC.

Xie, Y. (2025) *knitr: A general-purpose package for dynamic report generation in R* (version 1.50) [R package]. Available at: https://yihui.org/knitr/

Zeileis, A. & Grothendieck, G. (2005) zoo: S3 infrastructure for regular and irregular time series. *Journal of Statistical Software*, **14**, 1–27. https://doi.org/10.18637/jss.v014.i06

Zeileis, A. & Hothorn, T. (2002) Diagnostic checking in regression relationships. *R News*, **2**, 7–10. Available at: https://CRAN.R-project.org/doc/Rnews/

Zhu, H. (2024) *kableExtra: Construct complex table with "kable" and pipe syntax* (version 1.4.0) [R package]. CRAN. https://doi.org/10.32614/CRAN.package.kableExtra
